# Copper Regulates the Susceptibility of Zebrafish Larvae to Inflammatory Stimuli by Controlling Neutrophil/Macrophage Survival

**DOI:** 10.3389/fimmu.2019.02599

**Published:** 2019-11-08

**Authors:** MingYue Chen, Yi Luo, JiangPing Xu, Ming-Xian Chang, Jing-Xia Liu

**Affiliations:** ^1^Key Laboratory of Freshwater Animal Breeding, Ministry of Agriculture, College of Fisheries, Huazhong Agricultural University, Wuhan, China; ^2^State Key Laboratory of Freshwater Ecology and Biotechnology, Institute of Hydrobiology, Chinese Academy of Sciences, Wuhan, China

**Keywords:** copper, macrophages and neutrophils, phagocytosis, mROS, apoptosis, immune responses

## Abstract

Copper has been revealed to negatively affect the hematopoietic system, which has an important function in immune pathogen defense, but little is known about the potential mechanism. In this study, copper-stressed larvae exhibited significantly increased mortality as well as reduced percentages of GFP-labeled macrophages and neutrophils after *Aeromonas hydrophila* (*A. hydrophila*) infection. However, those copper-stressed GFP-labeled macrophages and neutrophils showed more rapid responses to *A. hydrophila* infection. The transcriptional profiles in copper-stressed macrophages or neutrophils were unveiled by RNA-Sequencing, and KEGG pathway analysis revealed enrichment of differentially expressed genes (DEGs) in lysosome, apoptosis, oxidative phosphorylation, phagosome, etc. The copper-stressed macrophages or neutrophils were revealed to have an increase in reactive oxygen species (ROS) and mitochondria ROS (mROS)-mediated apoptosis, and a reduction in phagocytosis. Furthermore, the *A. hydrophila-*infected copper-stressed macrophages or neutrophils were found to be unable to maintain a consistently increased expression in immune responsive genes. This study demonstrated for the first time that copper might induce the susceptibility of fish larvae to inflammatory stimuli *via* triggering macrophage or neutrophil apoptosis, leading to reduced phagocytic activities and non-sustainable immune responses in immune macrophages or neutrophils.

## Introduction

During zebrafish embryogenesis, primitive macrophages are initiated and specified at 12–16 hpf (hours post fertilization) and primitive neutrophils at 16 hpf (1, 2) from rostral blood island (RBI) (3, 4). Functional macrophages and neutrophils can be recruited to the infected sites and phagocyte invading bacteria as early as 30 and 52 hpf, respectively (5, 6). Zebrafish rely exclusively on innate immune system before they form functional adaptive immunity at around 28 dpf (days post fertilization) (6).

The neutrophils and macrophages, as hematopoietic leukocyte subsets in innate immune system in fish, form the first line of defense against the invasion of pathogens and the resultant diseases and mortalities. Recently, the zebrafish (*Danio rerio*) embryos and larvae have been proven to be unique vertebrate models for studying host–microbe interactions due to their advantages in *in vivo* imaging and genetic analysis (7, 8). However, to date, studies on host–microbe interaction in fish under a stressed environment are scarce, and little is known about the cellular biology and transcriptional responses of leukocyte cells to microbe infection in stressed fish.

*Aeromonas hydrophila*, a gram-negative opportunistic pathogen found in aquatic environment, is the main pathogen infecting farmed fish such as blunt snout bream (9), large yellow croaker (10), and gibel carp (*Carassiu auratus gibelio*) (11) in China. It has been found that the degradation of culture conditions, such as the increased stocking densities, poor water quality, etc., could increase the mortality rate of *A. hydrophila*-infected fish. However, the potential molecular mechanisms of *A. hydrophila* as an opportunistic pathogen in inducing fish mortality are still unknown.

It is widely known that excessive copper in aquatic environment can affect the development and survival of fish embryos and larvae (12–14). Copper also has a strong negative effect on the zebrafish hematopoietic system *via* reducing the hematopoiesis potential of head kidney (15), and excessive copper was associated with leukemia in human (16), suggesting the negative role of excessive copper in hematopoietic system and its immune response activity.

In this study, the gene expression profiles of GFP-positive cells in copper (Cu^2+^) stressed *lyz* and *mpx* transgenic larvae in which GFP labeling of neutrophils at 68 hpf and of those in *coro1a* transgenic larvae in which fluorescent GFP labeling of both neutrophils and macrophages at 68 hpf were revealed by RNA-Seq. Furthermore, the responses of copper-stressed larvae and their macrophages and neutrophils to *A. hydrophila* infection were further investigated, and the reactive oxygen species (ROS)-mediated apoptosis and the down-regulated phagocytic activities of neutrophils and macrophages were found to be associated with the sensitivity of copper-stressed fish larvae to *A. hydrophila* infection in this study.

## Materials and Methods

### Zebrafish Lines and Maintenance

Adult zebrafish, including AB, *Tg*(*coro1a*:eGFP) (7), *Tg*(*lyz*:eGFP) (17), and *Tg*(*mpx*:eGFP) (18) (the three transgenic lines were kindly provided by Professor Li Li, Southwest University, China), were raised in recirculating aquaculture systems (28 ± 0.5°C, 14:10 h light:dark), and were fed with hatched fairy shrimps three times each day. Male and female zebrafish were kept separately until mating and spawning. Eggs from the same batch (spawned by several pairs of adult fish) were collected, washed, and staged by morphological features based on the morphological criteria as described by Kimmel et al. (19) and then were used for different treatments and experiments as described below. The age of embryos and larvae was expressed in hours post fertilization (hpf) or days post fertilization (dpf).

### Chemical Treatment

In this study, 0.25 mg/L Cu^2+^ (3.9 μM) was used to stress the zebrafish embryos and larvae. NAC (*N*-acetylcysteine, Beyotime, Suzhou, China) and GSH (reduced glutathione, GBCBIO Tec. Inc., Guangzhou, China) were dissolved in DMSO in the dark as stock solutions (20). In experiments of co-exposure with ROS scavenger, embryos were pretreated with 200 μM NAC or 200 μM reduced GSH (21) at the fertilization stage, and when embryos developed to the sphere stage (4 hpf), Cu^2+^ (3.9 μM) was added separately to the pretreated medium.

### Tail Wounding

Embryos were collected and cultured in sterile pure water (egg water) instead of E3 water (5.0 mM NaCl, 0.17 mM KCl, 0.33 mM CaCl, 0.33 mM MgSO_4_, 0.05% methylene blue, pH ~7.4) to eliminate the influence from other ions in E3 medium as we performed previously (22–24). Larvae at 68 hpf from the Cu^2+^-stressed group and the control group without Cu^2+^ stress were wounded at tail fin with a sterile 24-gauge needle under a Stereoscopic Microscope (Leica M250FA) in fresh sterile filtered egg water with 0.02% tricaine as previously reported (7, 25). The wounded embryos were used for further analysis, such as mortality rate, *A. hydrophila* infection, and neutrophil/macrophage recruitment.

### Bacterial Challenge

In this study, *A. hydrophila* (isolated from blunt snout bream by assistant Professor Yi Luo) (26) was used to infect copper-stressed larvae at 68 hpf in two bath infection models: larvae were exposed to the bacteria in egg water by immersion only or immersion after injury. For the immersion only model, a trial test was performed first among Cu^2+^-stressed and no-Cu^2+^-stressed control larvae challenging with *A. hydrophila* infection beginning at 68 hpf to determine the mortalities at the concentrations of bacterial cells (10^6^ and 10^7^ cfu/ml), mimicking fish larvae under bacterial infection in a natural copper-stressed or no-copper-stressed environment. Over 50 embryos were used in each group and three biological replicates were performed in this test.

The wounded larvae of Cu^2+^-stressed or the control without Cu^2+^ stress were infected with *A. hydrophila* at 68 hpf *via* immersion post-injury model, and the migration dynamics of neutrophils/macrophages in the infected Cu^2+^-stressed and control larvae were analyzed separately at 0, 2, 4, and 6 hpi (hours post infection).

### Live Imaging

The wounded Cu^2+^-stressed and no-Cu^2+^-stressed control larvae after *A. hydrophila* infection were anesthetized and photographed separately using a Stereoscopic Microscope (Leica M250FA) with a fluorescent filter. *A. hydrophila*-infected and wounded larvae from Cu^2+^-stressed or control group (over 10 larvae/sample) were anesthetized and photographed at 0 hpi and then were cultured continuously under *A. hydrophila*-infected condition, and were further anesthetized and photographed at 2, 4, and 6 hpi separately. The numbers of GFP-labeled neutrophil/macrophage cells around the wounded locus (in red boxes) were counted and compared between Cu^2+^-stressed and no-Cu^2+^-stressed control larvae. In this study, red boxes for GFP-positive cell counting area were determined based on the wound loci in each larva as studies reported recently (7, 25), and the size of the red boxes was fixed for each larva in order to avoid the area variation for GFP^+^ cell counting.

### Quantitative Real-Time PCR (qRT-PCR) in Whole Embryos

Larvae were infected with *A. hydrophila* at 68 hpf, followed by collection at 6 hpi for testing the immune responses in the whole larvae. Briefly, 30–50 embryos/sample were used for total RNA extraction with TRizol Reagent (Ambion, USA), and cDNA was synthesized using an M-MLV Reverse-Transcript Kit (ABM Inc., Canada). Quantitative PCR was performed using iQ™ SYBR® Green Super mix (Bio-Rad Laboratories, USA) in a CFX Connect™ Real-Time PCR Detection System (Bio-Rad Laboratories, USA).

It has been reported that increased expression was observed in genes of *tlr5b* (9), *tnf*α, and *il1*β (27), and Toll-like receptor and JAK-STAT and MAPK pathways (10) in fish after *A. hydrophila* infection. Thus, in this study, the whole *A. hydrophila-*infected larvae were tested in the expression of the proinflammatory cytokine genes *il-6*, TLR signaling pathway genes (*tlr5b, jun, myd88, trif*, *IkBa, nfkbia, erk1/2, jnk)*, MAPK pathway genes (*dusp* and *rac1a*), and other immune and stress responsive genes (*stat1a, c3a, defb1*, and *hsp70.3*). Primer sequences for the aforementioned genes are shown in Table S1.

### RNA-Sequencing Analysis

*Tg*(*coro1a*:eGFP), *Tg*(*lyz*:eGFP), and *Tg*(*mpx*:eGFP) larvae from the Cu^2+^-stressed group and the no-Cu^2+^ control group at 68 hpf were collected and washed, and then were dissociated into single cells by 1 ml of homogenizer in solution of phosphate buffer saline (PBS) with 5% fetal bovine serum (FBS). Then, the homogenized cells were filtered through a 70 μm cell strainer as reported in literature (28, 29). Next, the GFP-positive cells (100–200 sorted cells/sample) of different lines from the Cu^2+^-stressed and control larvae were sorted separately by flow cytometry (FACS) (BD FacsAria SORP, 650110M3, BioDot, American) and the background fluorescence in cells from WT (wild type) fish without transgenic GFP was used as gate determination for GFP-positive cell sorting in this study.

The 100–200 sorted GFP-positive cells for each sample were then lysed in SMART-SeqTM V4 kit lysis buffer provided by Novogene Company. Next, the high-quality next-generation sequencing libraries for each sample (the sorted 100–200 sorted GFP cells) were constructed from as little as 10 pg total RNA by the Kit. Finally, the constructed libraries were sequenced using an Illumina HiSeqTM in Novogene Company.

Normalized read counts were used for fold change analysis, and fold changes were determined for each gene through dividing the number of tags in the normalized Cu^2+^-stressed libraries by that in the normalized control libraries by DEGSeq. Genes that were significantly altered as a result of the Cu^2+^ stresses (adjusted *P* < 0.05) were defined as differentially expressed genes (DEGs) and were used for further analysis. Pathway analysis and enriched KEGG pathways were conducted using KOBAS v2.0 based on the lists of DEGs (adjusted *P* < 0.05) for each of the treatments and lines. Hierarchical clustering was performed by TIGR Multi experiment Viewer (MeV) to generate different Heatmaps.

### Apoptosis Assay

*Tg*(*coro1a*:eGFP), *Tg*(*lyz*:eGFP), and *Tg*(*mpx*:eGFP) larvae from the Cu^2+^-stressed and control groups (100 larvae/sample) at 68 hpf were dissociated separately into single cells as mentioned above, and the dissociated single cells for each sample were separately used for Annexin V-PE and DAPI (4′,6-diamidino-2-phenylindole) (Beyotime Biotechnology) co-staining according to the instructions provided in the Annexin V-PE apoptosis assay kit (Beyotime Company, China). Then, the stained cells were used for analysis of cell apoptosis by FACS (CytoFLEX S, Beckman Coulter, USA). DAPI was used to distinguish dead vs. live cells. Additionally, propidium iodide (PI) staining for the aforementioned cells was used to distinguish the Annexin V-PE positive cells in terms of apoptosis, necroptosis, or pyroptosis in this study. For FACS analysis, background fluorescence was determined using the embryonic cells without reagent (Annexin V-PE, DAPI, or PI) staining. Three biological replicates were performed for each test, respectively, in this study.

### Phagocytosis Analysis

The aforementioned transgenic larvae from the Cu^2+^-stressed group and the no-Cu^2+^-stressed control group (100 larvae/sample) at 68 hpf were dissociated separately into single cells, followed by incubation with pHrodo™ Red BioParticles^@^ Conjugates Reagent (Molecular Probes, Life Technologies, Cat#P35361) at 28°C in the dark for over 30 min according to the manual instructions. The reagent is actually a kind of *E. coli* bioparticle that could be easily ingested by macrophages and neutrophils. The cells with uptake bacteria will exhibit red fluorescence, and the red fluorescence intensity depends on the surrounding acidic pH in cells. Next, the stained cells were used for analysis of red fluorescence intensity and percentage of red/GFP double-positive cells in the total GFP-positive cells by FACS (BD FacsAria SORP, 650110M3, BioDot, American), and the background fluorescence was determined using the samples that contain the pHrodo BioParticles but no macrophage and neutrophil cells.

In order to test the phagocytic activity of each macrophage or neutrophil in the control or copper-stressed embryos, the red and GFP double-positive cells were sorted separately from the Cu^2+^-stressed dissociated cells and the control, followed by collection and fixing with standard 2–4% paraformaldehyde (PFA) solution. Finally, the fixed red and GFP double-positive cells were smeared on slide glasses and observed under a Leica confocal microscope (Olympus FV1000 Confocal Microscope, Japan).

### ROS Detection

The aforementioned transgenic larvae from the Cu^2+^-stressed group and the control group (100 larvae/sample) at 68 hpf were dissociated separately into single cells as mentioned above, followed by incubation with the Fluorometric Intracellular ROS kit (Deep Red Fluorescence, Sigma-Aldrich, Cat# MAK142) according to the manual instructions. Finally, the isolated and stained cells were analyzed by FACS (CytoFLEX S, Beckman Coulter, USA). In this study, the FACS gate was determined based on the background fluorescence using a sample that contains the embryonic cells without the ROS Deep Red Fluorescence reagent staining.

### One Step Cell-Direct qRT-PCR

Larvae from the control and Cu^2+^-stressed groups were infected with *A. hydrophila* at 68 hpf, and the macrophages and neutrophils in both infected and uninfected larvae (100 larvae/sample) were sorted separately by FACS (BD FacsAria SORP, 650110M3, BioDot, American) at 6 or 24 hpi for cell direct quantitative real-time PCR (qRT-PCR) of immune and apoptosis genes. Briefly, the GFP-positive cells were sorted and lysed in solution provided by CellsDirect™ One-Step qRT-PCR Kit (Invitrogen, Cat#11753-100). Finally, the lysed solution was used as template for one step cell direct qRT-PCR using CellsDirect™ One-Step qRT-PCR Kit (Invitrogen) as reported previously (30, 31). Primer sequences for the genes tested in this study are shown in Table S1.

### Statistical Analysis

Larvae with different treatments were collected at the indicated stages as described above. The sample size and the biological replicates for each test were also mentioned above. Results of mortalities, FACS indicating phagocytosis, ROS, apoptosis, and qPCR were analyzed using one-way analysis of variance (ANOVA) and *post-hoc* Tukey's test on Statistic Package for Social Science (SPSS) 19.0 software. Comparison analyses of cell numbers around wounded locus and phagocytic *E. coli* number in each cell were performed using hypergeometric distribution in R-console software. Statistically significant differences between groups were indicated by ^***^P < 0.001, ^**^P < 0.01, ^*^P < 0.05, and ns, no significance.

## Results

### Increased Mortality Occurs in Copper-Stressed Larvae After *A. hydrophila* Infection

**Figure 1:**
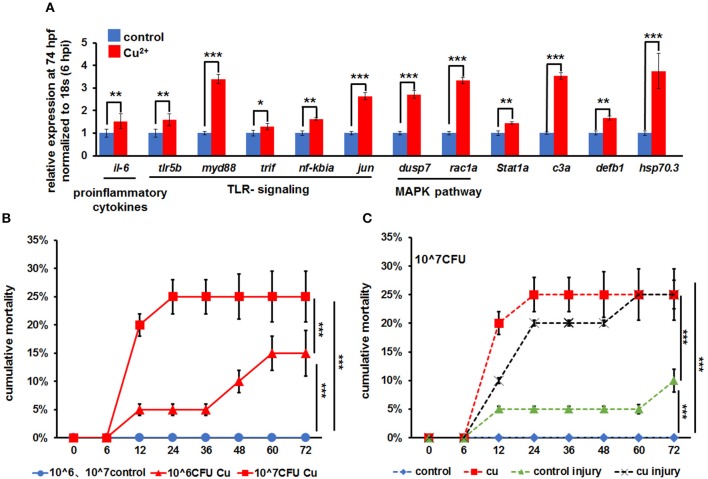
*A. hydrophila-*induced increased mortality in copper-stressed larvae. **(A)** *A. hydrophila* used in this study induced increased expression of immune-related genes in zebrafish larvae at 6 hpi (hours post infection), with increased expression observed in *il-6*, TLR signaling genes, MAPK signaling genes, *stat1a, c3a, defb1*, and *hsp70.3* after only immersion infection with *A. hydrophila*. **(B)** Larvae were immersed with a different dosage of *A. hydrophila* at 68 hpf (hours post fertilization), and cumulative mortalities of the no-copper-stressed control and the copper-stressed larvae were calculated separately at 6, 12, 24, 36, 48, 60, and 72 hpi. **(C)** Larvae were immersed only or immersed after injury with 10^7^ *A. hydrophila* at 68 hpf, and cumulative mortalities of the no-copper-stressed control and copper-stressed larvae were calculated separately at 6, 12, 24, 36, 48, 60, and 72 hpi. Three biological replicates were performed. ANOVA *post-hoc* Tukey's test. Data are presented as mean ± SD. ***P < 0.001; **P < 0.01; *P < 0.05.

The growth curve of *A. hydrophila* used in this study is shown in Figure S1. Larvae at 68 hpf were infected with *A. hydrophila* at the concentration of 10^6^ cfu/ml by immersion only. At 6 hpi, the whole larvae showed increased expression in proinflammatory cytokines *il-6*, TLR signaling pathway genes *tlr5b, jun, myd88, trif*, *IkBa, nfkbia, erk1/2, jnk*, MAPK pathway genes *dusp* and *rac1a*, and other immune and stress responsive genes like *stat1a, c3a, defb1*, and *hsp70.3* (Figure 1A).

Larvae from the control and Cu^2+^-stressed groups at 68 hpf were infected separately with *A. hydrophila* at concentrations of 10^6^ and 10^7^ cfu/ml. The Cu^2+^-stressed group showed a significant increase in mortality from 5.0 ± 0.5% to 25.0 ± 4.5% from 12 hpi to afterword (Figure 1B). Overall, the mortality in Cu^2+^-stressed larvae increased significantly with the increasing infection concentration of *A. hydrophila* (Figure 1B).

The mortalities of larvae in different groups were compared in terms of *A. hydrophila* infection manner: immersion only or immersion post injury. Specifically, in copper-stressed larvae, infection by immersion only with *A. hydrophila* at 10^7^ cfu/ml showed 20.0 ± 2.0% mortality at 12 hpi and 25.0 ± 4.5% at 24 hpi and later, in contrast to 0% in the control group. Meanwhile, infection by immersion post injury at 10^7^ cfu/ml exhibited 10.0 ± 0.5% to 25.0 ± 2.0% mortality in the Cu^2+^-stressed group at 12–72 hpi, which was significantly higher than that (5.0 ± 0.5% to 10.0 ± 2.0%) in the control injury group (Figure 1C).

### Copper-Stressed Neutrophils and Macrophages Respond Quickly to *A. hydrophila* Infection

At 68 hpf, the transgenic zebrafish larvae of *coro1a*-GFP, *lyz*-GFP, and *mpx*-GFP were infected with *A. hydrophila via* immersion only, and no obvious distribution change was observed in *coro1a*-GFP, *lyz*-GFP, or *mpx*-GFP cells in the larvae from the control and the copper-stressed groups at 24 hpi after *A. hydrophila* infection (Figures S2A–C).

**Figure 2:**
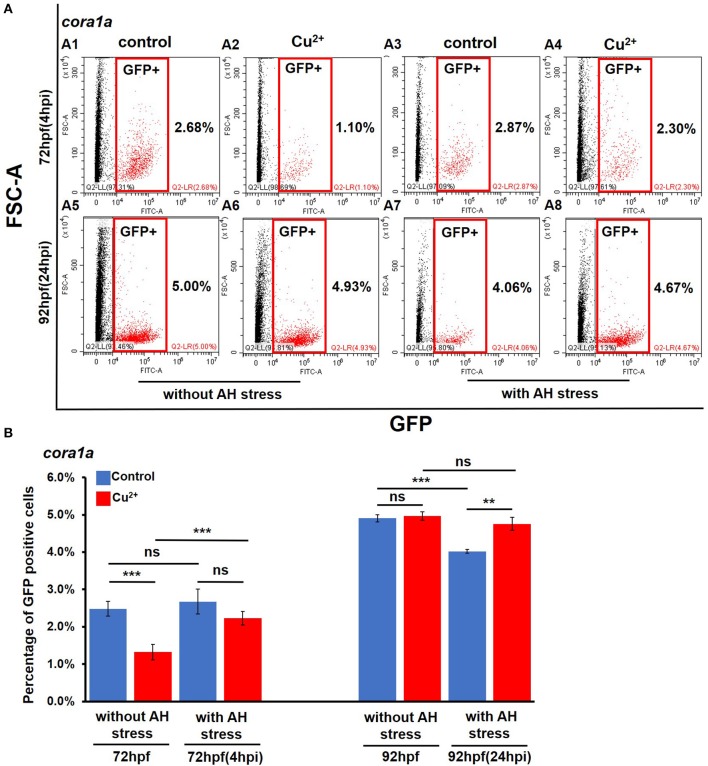
Percentages of *coro1a* promotor-driven GFP-positive cells in copper-stressed and the no-copper-stressed control larvae before and after *A. hydrophila* infection. FACS (flow cytometry) plots **(A1**–**A8)** (red boxes indicating GFP-positive cells) and percentages **(B)** of *coro1a* promotor-driven GFP-positive macrophages and neutrophils in copper-stressed and control larvae with or without *A. hydrophila* infection. Three biological replicates were performed. ANOVA *post-hoc* Tukey's test. Data are presented as mean ± SD. ***P < 0.001; **P < 0.01; and ns, no significance.

Under no *A. hydrophila* infection condition, compared with the control *coro1a* promotor-driven GFP transgenic larvae, the percentage of GFP-positive cells showed a significant decrease from 2.68 ± 0.20% to 1.52 ± 0.21% in Cu^2+^-stressed larvae (Figures 2A1,A2,B). Additionally, the Cu^2+^-stressed larvae rather than the control exhibited a significant increase in the percentage of neutrophil and macrophage cells at 4 h post *A. hydrophila* infection (Figures 2A1–A4,B). At 24 hpi, the percentage of GFP-positive cells showed no difference in Cu^2+^-stressed *coro1a* larvae (Figures 2A5–A8,B), in contrast to a significant reduction in the percentage of GFP-positive cells in the control larvae after *A. hydrophila* infection (Figures 2A5–A8,B). Furthermore, the percentages of GFP neutrophil cells in *lyz* transgenic larvae were significantly increased in Cu^2+^-stressed larvae at 4 h post both with and without *A. hydrophila* (72 hpf) infection (Figures S2D,E), and *A. hydrophila* infection significantly down-regulated the percentage of the *lyz*-GFP-positive cells in both the control and the Cu^2+^-stressed larvae at 92 hpf (24 hpi) (Figures S2D,E).

**Figure 3:**
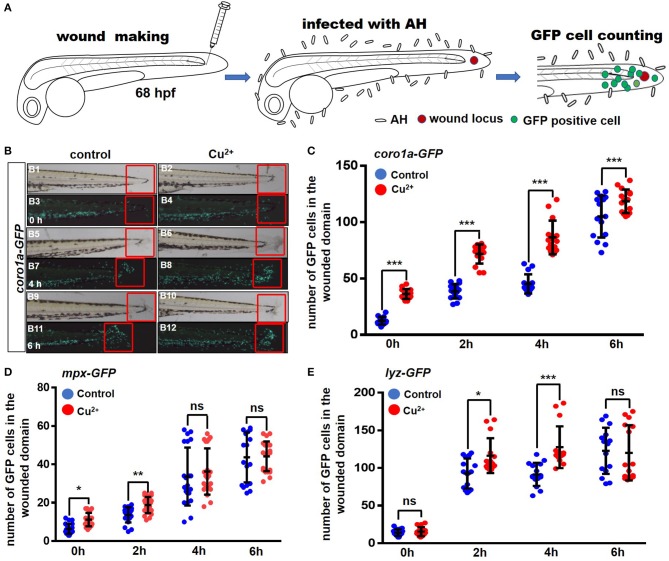
Copper-stressed macrophages and neutrophils respond more quickly to *A. hydrophila* infection. **(A)** Schematic view of tail injury, *A. hydrophila* infection, and GFP-positive cell counting, with immersion after injury being used in the assays. **(B)** The recruitment of GFP-positive macrophages and neutrophils around injury locus (in the fixed red boxes) were analyzed at 0, 2, 4, and 6 hpi, using *Tg* (*coro1a*:eGFP) transgenic fish. **(C–E)** Quantification analysis showed a significant increase in the number of GFP-positive cells in the area near the injury locus in the copper-stressed *Tg*(*coro1a*:eGFP) larvae compared with the no-copper-stressed control at 0, 2, 4, and 6 hpi **(C)**, in the copper-stressed *Tg* (*mpx*:eGFP) larvae **(D)**, and in the copper-stressed *Tg* (*lyz*:eGFP) larvae **(E)**. Three biological replicates were performed. Analysis with hypergeometric distribution in R-console software for bi-modal distribution to exhibit individual variations in each group in one experiment. Data are presented as mean ± SD. ***P < 0.001; **P < 0.01; *P < 0.05; and ns, no significance.

The infection model of immersion after injury was used to test the recruitment response of neutrophils and macrophages to *A. hydrophila* infection, and the experimental schematic is shown in Figure 3A. After marking both macrophages and neutrophils in zebrafish larvae with *Tg* (*coro1a*:eGFP) at 68 hpf, *coro1a*-GFP-positive cells were recruited around the wounded domain from 0 h post *A. hydrophila* infection (Figures 3B,C), and *coro1a*-GFP-positive cells in the Cu^2+^-stressed larvae responded more rapidly to *A. hydrophila* infection (Figures 3B,C) than the cells in the control larvae. Additionally, more *coro1a*-driven macrophage and neutrophil cells in Cu^2+^-stressed embryos were recruited to the infection and wounded domain from 0 to 6 hpi (Figures 3B,C).

Neutrophils driven by *lyz* or *mpx* promotor exhibited only slightly quicker responses in Cu^2+^-stressed larvae and were recruited to the infected locus more rapidly from 0 to 4 h post *A. hydrophila* infection, but exhibited no significant difference in larvae between the Cu^2+^-stressed and control groups at 6 hpi (Figures 3D,E and Figure S3).

### Transcriptional Profiles in Neutrophils and Macrophages

**Figure 4:**
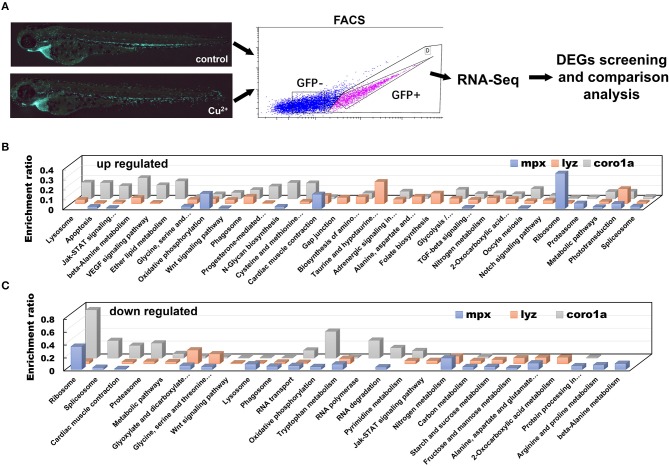
DEGs in copper-stressed *coro1a, mpx*, or *lyz* promotor-driven GFP-positive cells. **(A)** Schematic view of copper-stressed *coro1a, mpx*, or *lyz* promotor-driven GFP-positive cells as measured separately by FACS for RNA-Seq and comparison. **(B,C)** KEGG pathways over-represented for up-regulated **(B)** and down-regulated DEGs **(C)** of copper-stressed *coro1a, mpx*, and *lyz* promotor-driven GFP-positive cells, respectively, and the over-representation analysis was performed for all DEGs (adjusted *P* < 0.05) at 68 hpf.

Cell imaging observations suggest that more rapid recruitment responses of macrophage and neutrophil cells to the infection locus occurred in Cu^2+^-stressed larvae. In an effort to explore how copper influences the immune responses of neutrophil/macrophage to *A. hydrophila* infection, we further examined the transcriptional profiles of neutrophils and macrophages in copper-stressed embryos. The *coro1a*-, *mpx*-, or *lyz*-driven GFP-positive cells in the copper-stressed and control larvae at 68 hpf were sorted by FACS and used for RNA-seq (100–200 cells/sample) (Figure 4A). Over 27.7, 24.9, and 21.8 million sequences were obtained for Cu^2+^-stressed *coro1a, mpx*, or *lyz* driven GFP-positive cells, respectively, and 25.5, 31.5, and 24.5 million sequences for their control cells, respectively (Table S2). The error rate and GC content for RNA sequencing reads were determined, and the results are shown in Figure S4 (error rates) and Figure S5 (GC contents), respectively. The classifications of raw reads for different samples are shown in Figure S6. Moreover, results of FPKM distribution and Pearson correlation analysis are shown in Figures S7, S8, respectively, and the Pearson correlation values were 0.896, 0.928, and 0.885 for *coro1a, mpx*, or *lyz* cells, respectively.

DEGs were screened by DEGSeq and their Canoplot figures are shown as Cu^2+^ *vs* control for *coro1a* (Figure S9), *mpx* (Figure S10), and *lyz* cells (Figure S11), respectively. The overlapping DEGs for the *coro1a*-, *mpx*-, and *lyz*-positive GFP cells were then used for KEGG enrichment analysis. Enrichment was detected in lysosome, apoptosis, oxidative phosphorylation, phagosome, proteasome, etc. Additionally, Jak-STAT signaling, Wnt signaling, TGF-beta signaling, Notch signaling, and VEGF signaling were also enriched for the overlapping DEGs in all the three lines in copper-stressed larvae (Figures 4B,C and Tables S3–S8).

### Copper Down-Regulates Phagocytosis of Neutrophils and Macrophages

**Figure 5:**
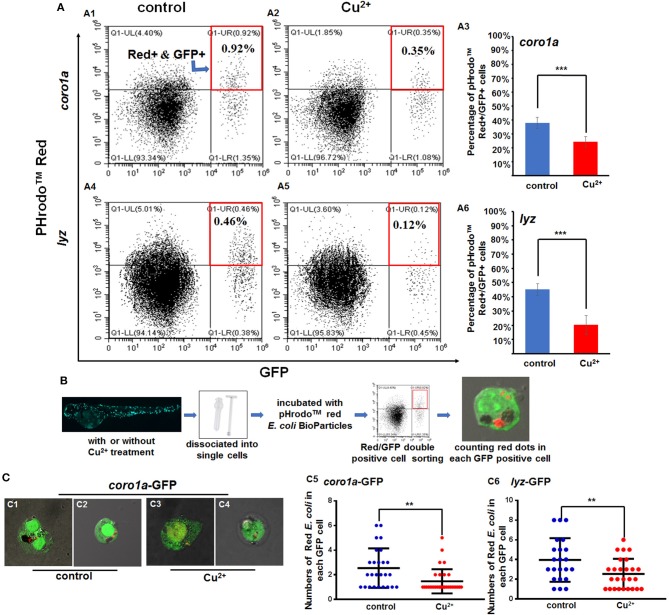
Phagocytosis of macrophages and neutrophils in copper-stressed and control larvae. **(A)** FACS plots **(A1,A2,A4,A5)** and percentages of red *E. coli*-positive cells in the total *coro1a* promotor-driven GFP-positive macrophage and neutrophil cells **(A3)** and in the total *lyz* promotor-driven GFP-positive neutrophil cells **(A6)**, respectively, following copper stimulation, red boxes indicating GFP and red positive cells by FACS. **(B)** Schematic view of *E. coli* phagocytosis activity tests for macrophages or neutrophils in copper-stressed or control larvae. **(C)** Mean (±SD) number of red *E. coli* cells per macrophage or neutrophil in the no-copper-stressed control or copper-stressed larvae. Macrophage with phagocytic red *E. coli* **(C1,C3)** or neutrophil cells **(C2,C4)** from the control **(C1,C2)** and copper-stressed larvae **(C3,C4)**, and mean (±SD) number of red *E. coli* cells per macrophage or neutrophil in *coro1a*-GFP cells **(C5)** or *lyz*-GFP cells **(C6)** was calculated. Two biological replicates were performed. Analysis with hypergeometric distribution in R-console software for bi-modal distribution to exhibit individual variations in each group in one experiment. ***P < 0.001; **P < 0.01.

Cell RNA-Seq results revealed the up-regulated expression of lysosome genes and the down-regulated expression of proteasome genes in copper-stressed *coro1a*-positive cells (Figures S12A,B), the down-regulated expression of both lysosome and phagosome genes in copper-stressed *mpx*-positive cells (Figures S12C,D), and the up-regulated expression of both lysosome and phagosome genes in copper-stressed *lyz*-positive cells (Figures S12E,F). Meanwhile, cell direct qRT-PCR detection showed the increased expression of the representative proteasome and lysosome genes in *coro1a*-, *lyz*-, and *mpx*-positive cells in copper-stressed larvae (Figure S12G). However, the percentage of PHrodo^TM^ red positive cells was down-regulated significantly from 37.95 ± 3.99% in the control *coro1a*-GFP-positive cells to 24.34 ± 3.75% in the copper-stressed *coro1a*-positive cells (Figures 5A1–A3), and the percentage of red/GFP double-positive cells was down-regulated significantly from 45.13 ± 4.00% to 20.18 ± 6.50% in the control and copper-stressed *lyz*-GFP-positive cells, respectively (Figures 5A4–A6).

Additionally, Red/GFP double-positive cells from the copper-stressed and the control larvae were sorted by FACS and smeared on slide glasses for confocal observation to test bacterial numbers in each neutrophil or macrophage cell (Figure 5B). In *coro1a*-GFP-labeled cells, ~3–6 red *E. coli-*positive dots were observed in the no-copper-stressed control neutrophils or macrophages cells (Figures 5C1,C2,C5) in contrast to about 1–2 red *E. coli-*positive dots observed in copper-stressed neutrophils or macrophages (Figures 5C3–C5), indicating that phagocytic activities were significantly down-regulated in copper-stressed neutrophil or macrophages (Figure 5C5). In *1yz*-GFP-labeled cells, ~2–6 red *E. coli-*positive dots were observed in control neutrophils (Figure 5C6), in contrast to about 1–3 red *E. coli-*positive dots observed in copper-stressed neutrophils (Figure 5C6).

### Copper Specifically Induces Neutrophil and Macrophage Apoptosis in Zebrafish Embryos

**Figure 6:**
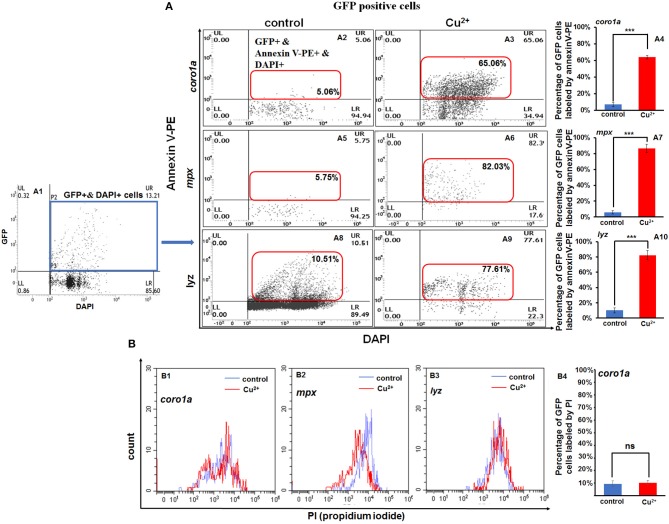
Increased apoptosis of macrophages and neutrophils in copper-stressed larvae. Annexin-V [**(A2–A10)**, in red boxes] and PI **(B1–B4)** labeling of apoptotic macrophages and neutrophils, and GFP-positive cells were selected for the analysis [**(A1)**, in blue box]. FACS panels **(A2,A3)** and percentage of apoptotic macrophages and neutrophils **(A4)** in copper-stressed *coro1a* promotor-driven GFP transgenic larvae; FACS panels **(A5,A6,A8,A9)** and percentages of apoptotic neutrophils in copper-stressed *mpx* transgenic larvae **(A7)** or *lyz* transgenic larvae **(A10)**. Three biological replicates were performed. ANOVA *post-hoc* Tukey's test. Data are presented as mean ± SD. ***P < 0.001; and ns, no significance.

Apoptosis signaling was enriched for the DEGs in copper-stressed *coro1a*-, *mpx*-, and *lyz*-positive GFP cells (Figure 4), and clustering was performed for DEGs in apoptosis signaling in copper-stressed neutrophil and macrophage cells (Figure S13). This enabled us to further investigate the mechanism of copper in regulating the embryonic susceptibility to bacterial infection by measuring the apoptosis of GFP-labeled macrophage and neutrophil cells with annexin V(red)/DAPI (blue) co-staining and PI staining. We found that *coro1a*-, *mpx*-, and *lyz*-driven GFP-positive cells all exhibited a significantly increased level of apoptosis in Cu^2+^-stressed larvae, with the percentages of labeled cells reaching 63.82 ± 2.20%, 86.55 ± 5.30%, and 82.30 ± 6.20%, in contrast to 6.78 ± 2.64%, 5.75 ± 2.12%, and 10.23 ± 3.25% in the control larvae, respectively (Figure 6A). PI staining was used to distinguish the annexin V red/GFP double-positive cells in terms of apoptosis, pyroptosis, or necroptosis in Cu^2+^-stressed larvae, with pyroptotic and necroptotic cells being annexin V and PI double-positive, and early apoptotic cells being annexin V positive but PI negative. No significant difference of PI level was observed between Cu^2+^-stressed and control larvae for all *coro1a*-, *lyz*-, and *mpx-*driven GFP-positive cells, and the percentages of PI positive cells were all around 10% (Figure 6B and Figure S14A).

However, GFP-negative cells in Cu^2+^-stressed larvae showed a slightly significant increase in annexin V-positive cells between Cu^2+^-stressed and control groups for the *coro1a, lyz*, and *mpx* transgenic lines, which was 3.53 ± 0.72%, 3.33 ± 0.43%, and 2.39 ± 0.53% in copper-stressed larvae vs. 0.04 ± 0.01%, 0.04 ± 0.01%, and 0.13 ± 0.09% in control larvae, respectively (Figures S14B,C).

### Copper-Induced ROS Triggers Neutrophil and Macrophage Apoptosis in Zebrafish Embryos

Cell RNA-Seq analysis revealed down-regulation of genes in oxidative phosphorylation in *coro1a* but up-regulation in both *mpx*- and *lyz*-positive cells in copper-stressed embryos (Figures S15A–C). Meanwhile, significant up-regulation was observed in the expression of oxidative phosphorylation genes, such as *cox4il, cox6c, cox8a, cox7b, atp6v0cb*, etc., in all *coro1a*-, *mpx*-, and *lyz*-positive cells in copper-stressed larvae in this study (Figure S15D).

**Figure 7:**
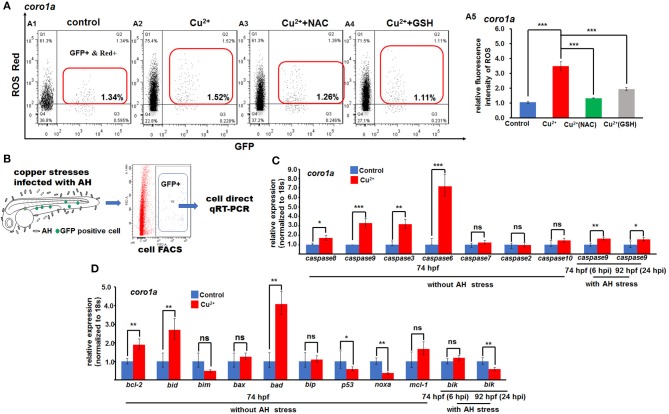
ROS- and mitochondrial ROS (mROS)-mediated apoptosis signaling in copper-stressed macrophages and neutrophils. ROS red labeling [**(A1–A4)**, in red boxes] and relative ROS fluorescence **(A5)** in macrophages and neutrophils in copper-stressed *coro1a*-driven GFP transgenic larvae. **(B)** Schematic view for testing the mROS-mediated apoptosis gene expression in copper-stressed macrophages and neutrophils *via* cell direct qRT-PCR. **(C)** Expression of different caspase genes in macrophages and neutrophils from copper-stressed *coro1a*-driven GFP transgenic larvae before and after *A. hydrophila* infection. **(D)** Expression of mROS-mediated apoptotic genes in aforementioned cells. Three biological replicates were performed. ANOVA *post-hoc* Tukey's test. Data are presented as mean ± SD. ***P < 0.001; **P < 0.01; *P < 0.05; and ns, no significance.

We further measured the ROS levels and the expression of functional ROS-triggered apoptosis genes in neutrophil and macrophage cells in copper-stressed larvae. The ROS level was ~3.3-fold higher in *coro1a*-positive neutrophil and macrophage cells (Figures 7A2,A5) than in the control (Figures 7A1,A5), and the increased ROS level in copper-stressed neutrophil and macrophage cells could be recovered to nearly normal level by ROS scavengers NAC and GSH (Figures 7A1–A5). Additionally, a significant increase was observed in the ROS levels in copper-stressed *mpx*- and *lyz*-positive cells, and ROS scavengers NAC could significantly down-regulate the ROS levels in those copper-stressed cells (Figure S16).

In this study, the expressions of ROS-mediated apoptosis signaling genes were further tested, and Figure 7B shows the schematic of cell direct qRT-PCR detection of apoptosis signaling genes in neutrophil and macrophage cells. The expression was significantly increased in *caspase8, caspase9, caspase3*, and *caspase6*, but normal in *caspase7, caspase2*, and *caspase10* in both copper-stressed *coro1a*-GFP-positive cells (Figure 7C) and *mpx-* and *lyz*-GFP-positive cells (Figures S17A1, S18A1). Additionally, genes *bcl-2, bid*, and *bad*, which function in mitochondrial ROS (mROS)-associated apoptosis signaling, exhibited significantly increased expression in copper-stressed *coro1a*-positive cells (Figure 7D). Moreover, a significant increase was observed in the expression of the aforementioned mROS-associated apoptosis signaling genes in both copper-stressed *mpx*-positive cells (Figure S17A2) and *lyz*-positive cells (Figure S18A2).

### Immune-Related Gene Expression in Copper-Stressed Neutrophils/Macrophages Cells

**Figure 8:**
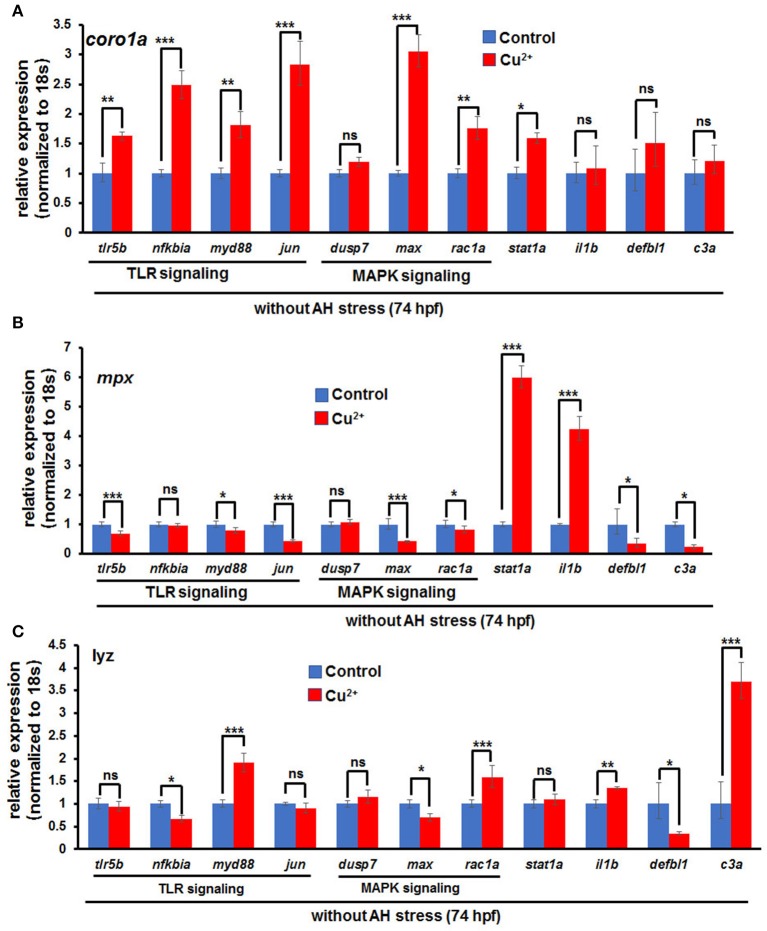
Expression of immune-related genes in copper-stressed macrophages and neutrophils under no *A. hydrophila* infection conditions. Expression of immune-related genes, including TLR signaling genes, MAPK signaling genes, *il1b, stat1a*, and *c3a*, in copper-stressed *coro1a*-GFP-positive cells **(A)**, *mpx*-GFP-positive cells **(B)**, and *lyz*-GFP-positive cells **(C)**. Three biological replicates were performed. ANOVA *post-hoc* Tukey's test. Data are presented as mean ± SD.***P < 0.001; **P < 0.01; *P < 0.05; and ns, no significance.

Cell RNA-Seq analysis revealed that Jak-STAT signaling, which has important function in immune responses to bacterial infection (10), was enriched for DEGs in copper-stressed *coro1a*-, *mpx*-, and *lyz*-positive cells (Figure 4). Thus, we examined further whether immune responses also contribute to the susceptibility of copper-stressed larvae to *A. hydrophila* infection. A significant up-regulation was found in the expression of heat shock responsive gene *hsp70.3*, and immune responsive genes, such as TLR signaling genes *myd88, jun, tlr5b*, and *nfkb1a*, MAPK pathway genes *max* and *rac1a*, and immune transcriptional factor *stat1a* in copper-stressed *coro1a* promotor-driven GFP-positive cells (Figure 8A). However, the expression was significantly down-regulated in several aforementioned genes, such as *myd88, jun, max*, or *rac1a* in either *mpx-* or *lyz*-positive cells from copper-stressed larvae (Figures 8B,C).

### Expression of Apoptosis and Immune-Related Genes in Copper-Stressed Neutrophils/Macrophage Cells After *A. hydrophila* Infection

The expression of the aforementioned genes in mitochondria-associated apoptosis signaling and in immune responses was further tested by cell direct qRT-PCR in copper-stressed neutrophil and macrophage cells after *A. hydrophila* infection. *Caspase9* still exhibited a slight increase of expression in *coro1a*-, *mpx*-, or *lyz*-positive cells in copper-stressed larvae at 6 and 24 hpi (Figure 7C and Figures S17A1, S18A1). However, apoptosis signaling gene *bik* exhibited no change or even down-regulation of expression in *coro1a*-, *mpx*-, and *lyz*-positive cells at 6 or 24 hpi (Figure 7D and Figures S17A2, S18A2).

**Figure 9:**
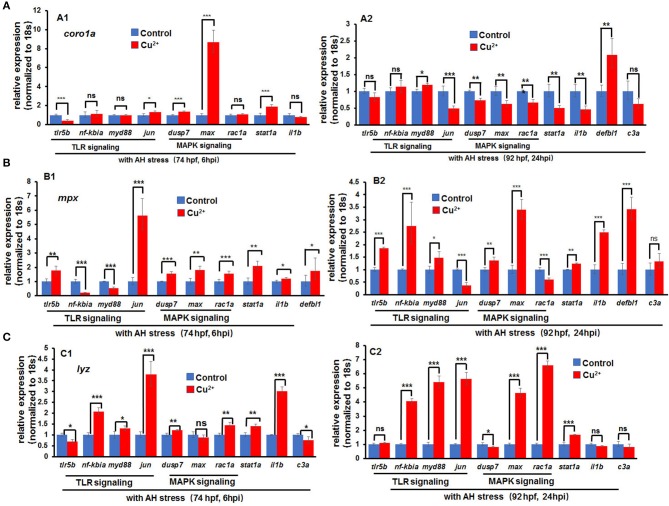
Expression of immune-related genes in copper-stressed macrophages and neutrophils after *A. hydrophila* infection. Expression of immune-related genes in copper-stressed *coro1a*-GFP-positive cells **(A)**, *mpx*-GFP-positive cells **(B)**, and *lyz*-GFP-positive cells **(C)** at 6 hpi **(A1–C1)** and 24 hpi **(A2–C2)**, respectively. Three biological replicates were performed. ANOVA *post-hoc* Tukey's test. Data are presented as mean ± SD. ***P < 0.001; **P < 0.01; *P < 0.05; and ns, no significance.

In copper-stressed *coro1a*-positive cells, the expression of the inflammatory cytokine *il1*β was down-regulated at 6 hpi and was significantly down-regulated at 24 hpi (Figures 9A1,A2). Additionally, the expression of TLR signaling pathway gene *tlr5b* was significantly down-regulated at 6 hpi (Figure 9A1), in contrast to a significant up-regulation in the expression of MAPK pathway genes *dusp7* and *max* (Figure 9A1), and a slight increase in the expression of *stat1a*, an important transcriptional factor in immune responses (Figure 9A1). At 24 hpi, the expression of TLR pathway genes *tlr5b* and *nf-kbia* exhibited no significant changes, but the signaling gene *jun* was significantly down-regulated (Figure 9A2). Moreover, MAPK pathway genes *dusp7, max*, and *rac1a* all exhibited significantly down-regulated expression, and so did *stat1a* (Figure 9A2).

The expression of the aforementioned genes was further tested in copper-stressed *mpx*- and *lyz*-positive cells. At 6 h post *A. hydrophila* infection, a significant up-regulation was observed in the expression of TLR pathway genes *tlr5b* as well as MAPK pathway genes *dusp7, max*, and *rac1a* in copper-stressed *mpx*-positive cells (Figure 9B1) and *lyz*-positive cells (Figure 9C1). An increased expression was also shown in the inflammatory cytokine *il1*β and immune transcriptional factor *stat1a* (Figures 9B1,C1). At 24 hpi, genes *jun* and *rac1a* exhibited significantly down-regulated expression in copper-stressed *mpx*-positive cells (Figure 9B2). Meanwhile, an increased expression was observed in TLR and MAPK pathway genes, inflammatory cytokine *il1*β, and immune transcriptional factor *stat1a* in copper-stressed *lyz*-positive cells (Figure 9C2).

## Discussion

*A. hydrophila* is a gram-negative opportunistic pathogen found in aquatic environment, but the potential molecular mechanisms of how it functions as an opportunistic pathogen in inducing fish mortality are still scare. Additionally, excess copper induces the degradation of culture condition as well as increased fish mortality and reduces fish hematopoiesis potentiality; however, the potential mechanisms and the mechanism linkages are still limited. This study, for the first time, not only linked the copper-induced culture condition degradation with *A. hydrophila*-induced increased mortality in fish larvae, but also demonstrated that the ROS-mediated apoptosis, the down-regulated phagocytic activities, and the non-sustainable immune responses occurred in macrophages and neutrophils together contributed to the sensitivity of copper-stressed fish larvae to *A. hydrophila* infection.

*A. hydrophila* was revealed to increase the expression of immune genes and induce mortality in zebrafish larvae, suggesting that *A. hydrophila* used in this study is a pathogenic bacterium for zebrafish larvae. A significant increase of mortality was observed in copper-stressed larvae even at *A. hydrophila* concentration of 10^6^ cfu/ml, and the mortality increased with the increasing infection concentration of *A. hydrophila*. Additionally, compared with injury, copper stress might be a more important contributor to *A. hydrophila-*induced mortality in zebrafish larvae. The integrated observations in this study indicate that metal copper, similar to other environmental factors or stressors, such as hypoxia stress (32), specific psychological stressors (33, 34), etc., could induce a significant increase in the susceptibility of vertebrates to pathogenic infection.

It was revealed that neutrophils and macrophages in copper-stressed embryos responded very quickly to *A. hydrophila* infection in this study, suggesting that copper might promote the responses of neutrophil and macrophage cells to bacteria-infected locus. mROS has been reported to link with macrophage-mediated bactericidal activity (35–37) and regulate neutrophil directional migration (25). In this study, the oxidative phosphorylation (OXPHOS) and ROS levels were observed to be up-regulated in neutrophil and macrophage cells in copper-stressed embryos, suggesting that high levels of oxidation stress might augment the rapid responses of the cells to the infected locus. Additionally, in this study, immune genes such as *il-6* and *il-1*β exhibited significantly increased expression in copper-stressed neutrophils and macrophages, which might also contribute to the rapid responses of the cells to *A. hydrophila* infection in copper-stressed zebrafish larvae. The points raised here are consistent with the report showing that ROS and *il-1*β act together in regulating neutrophil migration (25).

Additionally, the recruited differences of GFP-positive cells between *coro1a* and *mpx* and *lyz* lines indicate that neutrophils and macrophages might respond to *A. hydrophila* infection in different time windows, and neutrophils might be recruited to infected locus first, followed by macrophages in the *coro1a* transgenic line. These observations here are consistent with recent studies reporting that neutrophils are the first phagocytes to arrive at the inflammation locus as the major scavenger while macrophages emerge later to remove the cell debris and apoptotic cells (7, 38, 39).

However, phagocytic activities were significantly down-regulated in copper-stressed neutrophil and macrophage cells, as indicated by the significant reduction in both the percentage of red *E. coli* bioparticle positive cells in the total *coro1a-* or *lyz*-GFP-positive cells and the number of red *E. coli* bioparticles in each neutrophil or macrophage in copper-stressed larvae, despite increased expression of lysosome and phagosome genes observed in those cells. Neutrophils were the primary cells scavenging apoptotic bodies and small cell debris, in spite of their limited phagocytic capacity and rapid apoptosis (40). An activated macrophage could engulf more pieces of large cell debris, including apoptotic neutrophils (7). Thus, it could be deduced that the copper-stressed neutrophil and macrophage cells with reduced phagocytic activities could be recruited very quickly to the *A. hydrophila*-infected sites, but they could not effectively phagocyte the bacteria and remove the dead cells and cell debris, which might be a potential molecular mechanism underlying the copper-induced increased susceptibility to *A. hydrophila* infection for zebrafish larvae. However, it still remains unknown whether copper destroys the lysosome function in neutrophil and macrophage cells.

The percentage of apoptosis in neutrophils and macrophages showed a significant increase from around 5% in the control larvae to over 70% in copper-stressed larvae, in contrast to a slight increase in non-GFP-positive cells in copper-stressed larvae. These data suggest that copper might specifically induce high level of apoptosis in neutrophil and macrophage cells, which might be another contributor to the increased susceptibility of copper-stressed larvae to *A. hydrophila* infection. Copper-stressed neutrophil and macrophage cells showed that an increase in ROS and the mROS has been revealed to initiate apoptosis *via caspase8* or *caspase2*, and then trigger up-regulated expression of *bid, bcl2, bax bad, bim, caspase9*, etc (41, 42). Up-regulated expression was observed in *caspase8* and *caspase9* and annexin V-positive but PI-negative neutrophil and macrophage cells in copper-stressed neutrophil and macrophage cells, suggesting that the cells are apoptotic rather than necroptotic or pyroptotic. Moreover, the copper-stressed neutrophil and macrophage cells showed an increase in the expression of *caspase2, caspase8, bid, bax*, and *bad*, as well as their downstream *caspase9, caspase3, caspase6*, and *caspase7*, but a decrease in the expression of *noxa*. This implies that it is DNA damage or ROS that stimulates mitochondrion-linked apoptosis in copper-stressed embryos, which is consistent with a recent report summarizing the different cellular psychological stressors in stimulating apoptosis (42). Given the significantly increased mROS-mediated apoptosis specifically occurring in neutrophil and macrophage cells rather than non-neutrophil and macrophage cells, we demonstrated that copper-induced mROS might function more strikingly in innate immune cells in apoptosis compared with that in other cells, consisting with a point that mitochondria in neutrophils act a role restricted to apoptosis (43).

Furthermore, mROS has been reported to augment macrophage bactericidal activities (35–37), while in this study, with increased susceptibility to *A. hydrophila* infection, the copper-stressed neutrophils and macrophages showed an increase in ROS level, but a reduction in bactericidal activity. This implicates that copper might induce an extremely high level of ROS, leading to apoptosis. Additionally, the apoptosis in neutrophil and macrophage cells might destroy their phagocytic activities in copper-stressed larvae.

In this study, RNA-Seq (44) and cell direct qRT-PCR (30, 31) analyses revealed that the immune genes, such as proinflammatory cytokines, MAPK signaling genes, and TLR signaling genes, exhibited significantly increased expression in copper-stressed *coro1a, mpx*, and *lyz*, especially in *coro1a*-positive cells. It has been reported that ROS-induced signaling can provoke the synthesis of proinflammatory cytokines (45, 46), trigger MAPK signaling activity (47), and regulate the expression and localization of TLRs (48, 49). Thus, we speculate that the increased expression of the immune genes in the macrophages and neutrophils might respond to the elevated ROS occurred in the cells in this study.

However, after *A. hydrophila* infection, immune-related genes, such as *il-1*β, *stat1*, and MAPK signaling genes, including *dusp7, max*, and *rac1a*, could maintain up-regulated expression at 6 hpi, but exhibited significantly reduced expression at 24 hpi in *coro1a*-positive neutrophil and macrophage cells. These observations not only suggest that those neutrophil and macrophage cells in copper-stressed embryos were subject to excessive inflammation/infection and unable to resolve inflammation in a timely fashion, but also further convince that the activation of immune genes in copper-stressed macrophages and neutrophils resulted in elevated ROS rather than pathogen infection. It is well-known that ROS-induced MAPK and TLR/MyD88 signaling will lead to the activation of *caspase8* and *caspase3*, resulting in apoptosis in cells (47, 50). Thus, the ROS/MAPK and TLR/caspase-induced apoptosis might contribute to the inability of immune cells to respond effectively and sustainably to a subsequent bacterial challenge in copper-stressed larvae, which might further contribute to the increased susceptibility of copper-stressed larvae to *A. hydrophila* infection.

## Data Availability Statement

The datasets generated for this study are available on request to the corresponding author.

## Ethics Statement

All animals and experiments were conducted in accordance with the Guidelines for Experimental Animals approved by the Institutional Animal Care and Use Ethics Committee of Huazhong Agricultural University (permit number HZAUFI-2016-007).

## Author's Note

In this article, for the first time, we unveiled the transcriptional profiles of innate immune macrophage and neutrophil cells in fish larvae under environmental polluter copper stresses *via* RNA-Seq assays and linked the susceptibility of copper-stressed larvae to inflammatory stimuli with the reduced phagocytic activities and non-sustainable immune responses of the aforementioned cells. Mechanistically, we found that copper specifically induced macrophage and neutrophil apoptosis in the stressed larvae *via* inducing ROS and triggering mROS mediating apoptosis signaling in the cells. Briefly, in this study, we firstly demonstrated the potential molecular mechanisms underlying opportunistic pathogen in producing mortalities and diseases in fish under environmental polluter copper stresses and provided some inspiration to intensively study fish embryonic immune responses to opportunistic pathogens under stresses.

## Author Contributions

MC, JX, and J-XL designed the experiments and wrote the manuscript. YL provided the pathogen AH and helped to design the experiments. YL and M-XC helped in preparing the manuscript.

### Conflict of Interest

The authors declare that the research was conducted in the absence of any commercial or financial relationships that could be construed as a potential conflict of interest.
