# Supplementary material for: Copper Regulates the Susceptibility of Zebrafish Larvae to Inflammatory Stimuli by Controlling Neutrophil/Macrophage Survival
Source: Front Immunol. 2019 Nov 8;10:2599. doi: 10.3389/fimmu.2019.02599 (PMC6856049; doi:10.3389/fimmu.2019.02599)
Supplement: Supplementary file 1 [file Data_Sheet_1.PDF]

Table S1. The primers used for RT-PCR and qRT-PCR in this study.

Gene	Primer sequence	Product length	Gene Bank ID
TNF-α	F GCTGGATCTTCAAAGTCGGGTGTA<br>R TGTGAGTCTCAGCACACTTCCATC	139 bps	AB183467.1
IL-1β	F TGGACTTCGCAGCACAAAATG<br>R GTTCACTTCACGCTCTTGGATG	124 bps	NM_212844.2
IL-6	F AGACCGCTGCCTGTCTAAAA<br>R TTTGATGTCGTTCACCAGGA	136 bps	NM_001261449.1
TLR5b	F GCTATAATGCCATCTGC<br>R GTCCTGGTGTTTGTTGCT	168 bps	NM_001130595.2
myd88	F TCAGTTTGTCCACGAGAT<br>R CACCACCATCCTCTTACA	148 bps	NM_212814.2
TRIF	F TGGGTCAGTTTCCAAGTTCC<br>R CCACCTTCTGCCATTGTTTT	141 bps	EF204937.1
NF-kBIA	F GGGTTACTTGTCATTGTTGG<br>R TCAGGTGATAAGGCGTGT	191 bps	NM_199629.1
Jun	F CCGTGACACTGGCGATAA<br>R TAGAAGGCAAAGCGAAAC	176 bps	NM_199987.1
ERK1/2	F CCTGAACATGACCACACTGG<br>R TGCATCCCACAGACCAAATA	124 bps	NM_182888.2
JNK	F CAAACCTCTGCCAGGTCATT<br>R GCCGAAATCCAAAATCTTCA	176 bps	NM_131721.2
IκBa	F TTTCGGAGGAGATGGAGAGA<br>R CTGTTCAGGTACGGGTCGTT	184 bps	NM_213184.2
stat1a	F GGACGCTGAGCAAACCAT<br>R CACCTCGGACATCTGACTA	199 bps	NM_131480.1
β-actin	R CAACGGAAACGCTCATTGC<br>F GAGCCTGCCAGTCATATTTC	102 bps	AF057040.1
18S	F TTCCGACCGTAAACGATGC<br>R ACTCCTGGTGGTGCCCTTCC	155 bps	KY486501.1
cox6c	F GCTTTCCGTAGTCAGGC<br>R TGGGCAAATGAAACCTC	153 bps	NM_001204124.1
cox4i2	F ATTTTGCCGTCCCATCGT<br>R CTGCCTGTTCTATCACATCACC	158 bps	NM_200803.1
uqcrq	F TTTGTGGAGGCGATTTAG<br>R GTCAGCGGGATTCTTCTT	121 bps	NM_001002495.3
cox7b	F GAACCTCAGCGGTCAAAG<br>R AATGTGGCTCCAGCAATC	111 bps	NM_001130602.1
cox8a	F CGGTATCACTTTCCTCTTCC<br>R CAGAGCGGCTGTTGTCTC	194 bps	NM_001303053.1
atp5ia	F AGACGCTGAAATCAAAGG<br>R CCCATAAATCAACCCAAT	164 bps	NM_001172637.1

lgmn	F TGAAATCCACGCTCATCT<br>R AGTGTCTGACGGCTGTTT	158 bps	NM_214759.1
arsa	F AATCGCCCATTCTTCTTG<br>R AATCTTCCCTACAGTGCC	138 bps	NM_001013543.2
lamp1	F GAGTCGGACCGTGCATCT<br>R AGCGTTCCCACCAAGTAG	182 bps	NM_001326532.1
gnsa	F TAACATCGCCAAGAGCAT<br>R ACCTGAACCGTGAGTCGT	122 bps	NM_001030208.1
prdx1	F CTTGAGCACGACCTATTT<br>R AACTGTCCATCAGGCATC	165 bps	NM_001013471.2
atp6v0cb	F TCAGCAGCGATGGTCTTC<br>R AGCGATTAGCACAGCCAC	174 bps	NM_205554.1
hsp70.3	F TACAGCGGAAAGCGAGAC<br>R AGGTCAATGCCAATAGCG	145 bps	NM_131397.3
ptpn5	F GCTGTCCCATTATCGTCA<br>R TCCTTAGGCTGTAGTCATCT	144 bps	FN428702.1
psme1	F TGGGATAAGACATTACACG<br>R AACTGTTCAGCCTCCTTG	172 bps	NM_131375.2
max	F CGAAGAATGAGCGACAA<br>R ATCCCGAAGGCTGTGAA	150 bps	NM_131220.1
rac1a	F CGGAGGTCAGACATCAT<br>R GTTGGGTTCAGGCAGAG	192 bps	NM_199771.1
c3a.1	F TGCTATGCAAGAAGGGAG<br>R AGACGGCTTTATTGAGTT	173 bps	NM_131242.1
cbl	F ACAGTGGGCTATCGGTTAC<br>R GTCTTGAGGTGAGGGTTGA	175 bps	NM_001007330.1
defbl1	F TGCCTTCTGAACTTTACT<br>R CATTTCTGTCCCATTTATC	171 bps	NM_001081553.1
dusp7	F GGATGCAAAGCCTTCTACCT<br>R CATCAGAACAATCCGAACTA	160 bps	NM_199774.3
tal1	F GCGGGAGTCTGCTGGAT<br>R AGCGGTAGGCTGGAATG	112 bps	NM_213237.1
lmo1	F GCACGAAGACTGCCTGAA<br>R CCGTTGTACCAAAGAGCC	125 bps	NM_173219.3
bik	F GGAAGAATCCTCGCTAA<br>R GATGCCAGGGTGTATGT	213 bps	NM_001045038.2
bad	F GATGAATGAGGAGGACTGAC<br>R TCCAAAGAAATGCCAACC	233 bps	AF231017.2
bax	F TGTATGAGCGTGTTCGTCG<br>R CTGGTTGAAATAGCCTTGATG	304 bps	AF231015.1
bid	F AATGGTGAATCGGGAAGG<br>R CTGTAGGTCGCTGGTGGA	113 bps	NM_001079826.1
noxa1	F GGTTGCGTCCACTCACATCG<br>R ATCCCTCCTCAAGCCTGCGG	117 bps	NM_001030100.2

bim	F GAGCCGCAAGACGGACAAA<br>R CAGTTCACGAGCGACCACC	117 bps	EF539840.1
bcl-2	F GTCACTCGTTCAGACCCTCAT<br>R GACGCTTTCCACGCACAT	237 bps	AY695820.1
bip	F ACCCGCCACAGCCTACCAGA<br>R TGCGATGCGTCCCACAAGC	157 bps	AF317837.1
mcl-1	F CCGACGATGAAGCGAGTG<br>R AAAGACCAGGACGGACAACAG	370 bps	NM_131599.1
casp2	F TCGGGCATTTAGCACCTT<br>R AAGCCTGTGGTCTGTGGG	260 bps	NM_001042695.1
casp3	F TGTTCTTTATTCAGGCTTGTC<br>R TCACTGCCATACTTTGTCATC	223 bps	NM_131877.3
casp6	F GGATTGGGCTACCGTTCT<br>R GTCCATCGGAGTCACAGG	351 bps	NM_001020497.1
casp7	F CCGCAAAGGGAGATTTAGGC<br>R CAGGAGCAGTCGCTGTGGTC	339 bps	NM_001020607.1
casp8	F CCTGATTCTGCGACTGGA<br>R TAGGCTGAGACACCTTTACG	171 bps	NM_131510.2
casp9	F GATTCTTCAGCGGCACA<br>R CTCCAGGTCTTTCACCAGTT	163 bps	NM_001007404.2
casp10	F ACCCAGCGAGCAGTGTTACG<br>R CCAGGTTCCCTTGGATTTGT	165 bps	MG958003.1

Table S2. Classification of raw reads for sample of *coro1a* control-68hpf, *coro1a* Cu-68hpf, *mpx* control-68hpf, *mpx* Cu-68hpf, *lyz* control-68hpf, *lyz* Cu-68hpf

sample	Clean reads	Containing N	Low Quality	Adapter Related	PCR Primer Contaminated
coro1a control-68hpf	25453926<br>82.27%	0.1<br>0.00%	3728568<br>12.05%	354126<br>1.14％	1402980<br>4.53%
coro1a cu-68hpf	27729226<br>91.16%	0.1<br>0.00%	1921540<br>6.32%	253511<br>0.83%	514879<br>1.69%
mpx control-68hpf	24531692<br>87.60%	0.1,<br>0.00%	2712458<br>9.69%	179670<br>0.64%	578976<br>2.07%
mpx cu-68hpf	24855294<br>86.21%	0.1<br>0.00%	2778861<br>9.64%	321212<br>1.11%	876207<br>3.04%
lyz control-68hpf	31498799<br>82.40%	617182<br>1.61%	4037548<br>10.56%	1678929<br>4.39%	395228<br>1.03%
lyz cu-68hpf	21750048<br>79.96%	867760<br>3.19%	3155388<br>11.60%	1095740<br>4.03%	332159<br>1.22%

Table S3. Pathway analysis results of genes with reduced expression in *coro1a-gfp* embryos exposed to Cu^{2+} at 68 hpf (hours post fertilization).

#Column "Pathway ID" stands for Pathway identifiers used in KEGG

#Column "Term" stands for the definition of the Pathway ID

#Column "P value" stands for the enrichment p-value of the Pathway ID as calculated by the Fisher's exact test

#Column "Enrichment Score" stands for the Enrichment Score value of the Pathway ID, which equals "(-log10(P-value))"

#Column "Corrected P-Value" stands for the false discovery rate of the Pathway ID as estimated by the Benjamini & Hochberg method

#Column "Input number" stands for the Count of the DE genes' entities directly associated with the listed Pathway ID

#Column "Background number" stands for the total number of the DE genes' entities

#Column " Enrichment ratio " equals "input number/ background number"

Pathway ID	Term	P value	Enrichment score	Corrected P-Value	Input number	Background number	Enrichment ratio
dre03010	Ribosome	1.36E-83	82.87	1.08E-81	107	135	0.792592593
dre00190	Oxidative phosphorylation	2.37E-34	33.63	9.36E-33	60	145	0.413793103
dre03040	Spliceosome	1.55E-16	15.81	4.09E-15	37	136	0.272058824
dre04260	Cardiac muscle contraction	3.68E-07	6.43	7.26E-06	19	97	0.195876289
dre03050	Proteasome	5.28E-06	5.28	8.34E-05	13	56	0.232142857
dre01100	Metabolic pathways	4.27E-05	4.37	0.000561625	87	1269	0.06855792
dre03020	RNA polymerase	0.000127	3.9	0.00137267	8	29	0.275862069
dre03013	RNA transport	0.000143	3.84	0.00137267	19	155	0.122580645
dre03018	RNA degradation	0.000156	3.81	0.00137267	13	81	0.160493827
dre00240	Pyrimidine metabolism	0.003921	2.41	0.030973785	12	105	0.114285714
dre04623	Cytosolic DNA-sensing pathway	0.011177	1.95	0.080274716	6	39	0.153846154
dre03008	Ribosome biogenesis in eukaryotes	0.028835	1.54	0.189830603	8	78	0.102564103
dre00830	Retinol metabolism	0.04384	1.36	0.266415173	5	41	0.12195122
dre00360	Phenylalanine metabolism	0.05027	1.3	0.283668971	3	17	0.176470588
dre03015	mRNA surveillance pathway	0.055548	1.26	0.29255327	8	90	0.088888889
dre00790	Folate biosynthesis	0.063853	1.19	0.31527529	3	19	0.157894737
dre00350	Tyrosine metabolism	0.06966	1.16	0.323712816	4	33	0.121212121
dre00230	Purine metabolism	0.082430	1.08	0.361779583	13	187	0.069518717
dre03022	Basal transcription factors	0.136468	0.86	0.560191829	4	43	0.093023256
dre03440	Homologous recombination	0.141820	0.85	0.560191829	3	28	0.107142857
dre00232	Caffeine metabolism	0.228510	0.64	0.859634444	1	5	0.2
dre00400	Phenylalanine, tyrosine and tryptophan biosynthesis	0.261167	0.58	0.867986356	1	6	0.166666667
dre00982	Drug metabolism - cytochrome P450	0.26175	0.58	0.867986356	3	39	0.076923077
dre03060	Protein export	0.269969	0.57	0.867986356	2	22	0.090909091
dre03430	Mismatch repair	0.285879	0.54	0.867986356	2	23	0.086956522
dre05168	Herpes simplex infection	0.295456	0.53	0.867986356	10	182	0.054945055

Table S4. Pathway analysis results of genes with increased expression in *coro1a-gfp* embryos exposed to Cu^{2+} at 68 hpf (hours post fertilization).							
#Column "Pathway ID" stands for Pathway identifiers used in KEGG							
#Column "Term" stands for the definition of the Pathway ID							
#Column "P value" stands for the enrichment p-value of the Pathway ID as calculated by the Fisher's exact test							
#Column "Enrichment Score" stands for the Enrichment Score value of the Pathway ID, which equals “(-log10(P-value))”							
#Column "Corrected P-Value" stands for the false discovery rate of the Pathway ID as estimated by the Benjamini & Hochberg method							
#Column "Input number" stands for the Count of the DE genes' entities directly associated with the listed Pathway ID							
#Column "Background number" stands for the total number of the DE genes' entities							
#Column " Enrichment ratio " equals “input number/ background number”							
Pathway ID	Term	P value	Enrichment score	Corrected P-Value	Input number	Background number	Enrichment ratio
dre04142	Lysosome	0.000180425	3.743703815	0.023816071	24	144	0.166666667
dre04210	Apoptosis	0.002471501	2.607039263	0.163119046	16	98	0.163265306
dre00410	beta-Alanine metabolism	0.012682542	1.896793704	0.464480105	7	33	0.212121212
dre04630	Jak-STAT signaling pathway	0.015433938	1.811523243	0.464480105	16	122	0.131147541
dre04370	VEGF signaling pathway	0.022682625	1.644306682	0.464480105	12	86	0.139534884
dre00565	Ether lipid metabolism	0.026160242	1.582358238	0.464480105	7	39	0.179487179
dre04914	Progesterone-mediated oocyte maturation	0.027607005	1.558980708	0.464480105	14	110	0.127272727
dre00510	N-Glycan biosynthesis	0.028150309	1.550516827	0.464480105	8	49	0.163265306
dre00270	Cysteine and methionine metabolism	0.043049214	1.366034773	0.631388473	7	44	0.159090909
dre00500	Starch and sucrose metabolism	0.055943411	1.252251057	0.665725123	7	47	0.14893617
dre00280	Valine, leucine and isoleucine degradation	0.065740046	1.182169994	0.665725123	7	49	0.142857143
dre00380	Tryptophan metabolism	0.065740046	1.182169994	0.665725123	7	49	0.142857143
dre00562	Inositol phosphate metabolism	0.069843481	1.155874124	0.665725123	9	71	0.126760563
dre00330	Arginine and proline metabolism	0.074336344	1.128798801	0.665725123	9	72	0.125
dre00053	Ascorbate and aldarate metabolism	0.081753817	1.087491959	0.665725123	4	22	0.181818182
dre00563	Glycosylphosphatidylinositol(GPI)-anchor biosynthesis	0.081753817	1.087491959	0.665725123	4	22	0.181818182
dre00564	Glycerophospholipid metabolism	0.085737326	1.066830064	0.665725123	11	97	0.113402062
dre04114	Oocyte meiosis	0.109638618	0.960036447	0.772990637	14	138	0.101449275
dre00511	Other glycan degradation	0.11199552	0.950799349	0.772990637	4	25	0.16
dre04540	Gap junction	0.119221528	0.923645317	0.772990637	13	128	0.1015625
dre04110	Cell cycle	0.122975783	0.910180403	0.772990637	14	141	0.09929078
dre04621	NOD-like receptor signaling pathway	0.130620312	0.883989284	0.783721871	6	48	0.125
dre00480	Glutathione metabolism	0.147992195	0.82976119	0.830943955	6	50	0.12
dre00600	Sphingolipid metabolism	0.15259141	0.816469914	0.830943955	7	62	0.112903226
dre04145	Phagosome	0.175171458	0.756536655	0.830943955	15	164	0.091463415

Table S5. Pathway analysis results of genes with reduced expression in *mpx-gfp* embryos exposed to Cu^{2+} at 68 hpf (hours post fertilization).

#Column "Pathway ID" stands for Pathway identifiers used in KEGG

#Column "Term" stands for the definition of the Pathway ID

#Column "P value" stands for the enrichment p-value of the Pathway ID as calculated by the Fisher's exact test

#Column "Enrichment Score" stands for the Enrichment Score value of the Pathway ID, which equals "(-log10(P-value))"

#Column "Corrected P-Value" stands for the false discovery rate of the Pathway ID as estimated by the Benjamini & Hochberg method

#Column "Input number" stands for the Count of the DE genes' entities directly associated with the listed Pathway ID

#Column "Background number" stands for the total number of the DE genes' entities

#Column " Enrichment ratio " equals "input number/ background number"

Pathway ID	Term	P value	Enrichment score	Corrected P-Value	Input number	Background number	Enrichment ratio
dre04142	Lysosome	8.49E-05	4.07	0.007726456	12	144	0.083333333
dre03013	RNA transport	0.006548991	2.18	0.169018577	9	155	0.058064516
dre04141	Protein processing in endoplasmic reticulum	0.006561488	2.18	0.169018577	10	184	0.054347826
dre00910	Nitrogen metabolism	0.007429388	2.13	0.169018577	3	17	0.176470588
dre00250	Alanine, aspartate and glutamate metabolism	0.013156754	1.88	0.239452923	4	41	0.097560976
dre00330	Arginine and proline metabolism	0.020535796	1.69	0.284149285	5	72	0.069444444
dre00380	Tryptophan metabolism	0.022799268	1.64	0.284149285	4	49	0.081632653
dre04145	Phagosome	0.024980157	1.6	0.284149285	8	164	0.048780488
dre00410	beta-Alanine metabolism	0.036671908	1.44	0.370793733	3	33	0.090909091
dre00310	Lysine degradation	0.052453787	1.28	0.427070089	4	65	0.061538462
dre03030	DNA replication	0.053984493	1.27	0.427070089	3	39	0.076923077
dre00830	Retinol metabolism	0.060483332	1.22	0.427070089	3	41	0.073170732
dre00071	Fatty acid degradation	0.067329818	1.17	0.427070089	3	43	0.069767442
dre00790	Folate biosynthesis	0.067693992	1.17	0.427070089	2	19	0.105263158
dre04150	mTOR signaling pathway	0.075329971	1.12	0.427070089	4	74	0.054054054
dre03060	Protein export	0.085529743	1.07	0.427070089	2	22	0.090909091
dre00190	Oxidative phosphorylation	0.089378778	1.05	0.427070089	6	145	0.04137931
dre00280	Valine, leucine and isoleucine degradation	0.089839164	1.05	0.427070089	3	49	0.06122449
dre00510	N-Glycan biosynthesis	0.089839164	1.05	0.427070089	3	49	0.06122449
dre00480	Glutathione metabolism	0.093861558	1.03	0.427070089	3	50	0.06
dre01200	Carbon metabolism	0.12164618	0.91	0.527133448	5	123	0.040650407
dre00760	Nicotinate and nicotinamide metabolism	0.131544998	0.88	0.53244864	2	29	0.068965517
dre00524	Butirosin and neomycin biosynthesis	0.134574931	0.87	0.53244864	1	6	0.166666667
dre04514	Cell adhesion molecules (CAMs)	0.158896863	0.8	0.564218872	5	135	0.037037037
dre00630	Glyoxylate and dicarboxylate metabolism	0.159863875	0.8	0.564218872	2	33	0.060606061
dre00020	Citrate cycle (TCA cycle)	0.167110462	0.78	0.564218872	2	34	0.058823529
dre03320	PPAR signaling pathway	0.177038627	0.75	0.564218872	3	68	0.044117647
dre04110	Cell cycle	0.179079129	0.75	0.564218872	5	141	0.035460993
dre02010	ABC transporters	0.181764928	0.74	0.564218872	2	36	0.055555556

Table S6. Pathway analysis results of genes with increased expression in *mpx-gfp* embryos exposed to Cu^{2+} at 68 hpf (hours post fertilization).

#Column "Pathway ID" stands for Pathway identifiers used in KEGG

#Column "Term" stands for the definition of the Pathway ID

#Column "P value" stands for the enrichment p-value of the Pathway ID as calculated by the Fisher's exact test

#Column "Enrichment Score" stands for the Enrichment Score value of the Pathway ID, which equals "(-log10(P-value))"

#Column "Corrected P-Value" stands for the false discovery rate of the Pathway ID as estimated by the Benjamini & Hochberg method

#Column "Input number" stands for the Count of the DE genes' entities directly associated with the listed Pathway ID

#Column "Background number" stands for the total number of the DE genes' entities

#Column " Enrichment ratio " equals "input number/ background number"

Pathway ID	Term	P value	Enrichment score	Corrected P-Value	Input number	Background number	Enrichment ratio
dre03010	Ribosome	4.88E-52	51.31	1.56E-50	48	135	0.355555556
dre00190	Oxidative phosphorylation	6.77E-17	16.17	1.08E-15	22	145	0.151724138
dre04260	Cardiac muscle contraction	7.05E-11	10.15	7.52E-10	14	97	0.144329897
dre03050	Proteasome	0.035564484	1.45	0.284515869	3	56	0.053571429
dre01100	Metabolic pathways	0.068860543	1.16	0.440707477	22	1269	0.017336485
dre04744	Phototransduction	0.093409594	1.03	0.498184499	2	40	0.05
dre04141	Protein processing in endoplasmic reticulum	0.199167586	0.7	0.775834341	4	184	0.02173913
dre03040	Spliceosome	0.242452506	0.62	0.775834341	3	136	0.022058824
dre03060	Protein export	0.24699813	0.61	0.775834341	1	22	0.045454545
dre03018	RNA degradation	0.270364975	0.57	0.775834341	2	81	0.024691358
dre03440	Homologous recombination	0.300773642	0.52	0.775834341	1	28	0.035714286
dre04060	Cytokine-cytokine receptor interaction	0.325651032	0.49	0.775834341	3	161	0.01863354
dre04210	Apoptosis	0.347109431	0.46	0.775834341	2	98	0.020408163
dre04672	Intestinal immune network for IgA production	0.382051562	0.42	0.775834341	1	38	0.026315789
dre04623	Cytosolic DNA-sensing pathway	0.389643931	0.41	0.775834341	1	39	0.025641026
dre00260	Glycine, serine and threonine metabolism	0.440257666	0.36	0.775834341	1	46	0.02173913
dre03420	Nucleotide excision repair	0.440257666	0.36	0.775834341	1	46	0.02173913
dre04621	NOD-like receptor signaling pathway	0.453937022	0.34	0.775834341	1	48	0.020833333
dre00510	N-Glycan biosynthesis	0.46065164	0.34	0.775834341	1	49	0.020408163
dre04120	Ubiquitin mediated proteolysis	0.510067469	0.29	0.81610795	2	137	0.01459854
dre04115	p53 signaling pathway	0.599339803	0.22	0.908515449	1	73	0.01369863
dre05168	Herpes simplex infection	0.66234019	0.18	0.908515449	2	182	0.010989011
dre04350	TGF-beta signaling pathway	0.702540112	0.15	0.908515449	1	97	0.010309278
dre05132	Salmonella infection	0.716961431	0.14	0.908515449	1	101	0.00990099
dre04620	Toll-like receptor signaling pathway	0.723909439	0.14	0.908515449	1	103	0.009708738
dre04630	Jak-STAT signaling pathway	0.782018679	0.11	0.908515449	1	122	0.008196721

dre04114	Oocyte meiosis	0.821405805	0.09	0.908515449	1	138	0.007246377
dre04110	Cell cycle	0.827961311	0.08	0.908515449	1	141	0.007092199
dre04810	Regulation of actin cytoskeleton	0.851392524	0.07	0.908515449	2	271	0.007380074
dre04144	Endocytosis	0.87163518	0.06	0.908515449	2	286	0.006993007
dre04310	Wnt signaling pathway	0.884618611	0.05	0.908515449	1	173	0.005780347
dre04010	MAPK signaling pathway	0.908515449	0.04	0.908515449	2	320	0.00625

Table S7. Pathway analysis results of genes with reduced expression in *lyz-gfp* embryos exposed to Cu^{2+} at 68 hpf (hours post fertilization).							
#Column "Pathway ID" stands for Pathway identifiers used in KEGG							
#Column "Term" stands for the definition of the Pathway ID							
#Column "P value" stands for the enrichment p-value of the Pathway ID as calculated by the Fisher's exact test							
#Column "Enrichment Score" stands for the Enrichment Score value of the Pathway ID, which equals "(-log10(P-value))"							
#Column "Corrected P-Value" stands for the false discovery rate of the Pathway ID as estimated by the Benjamini & Hochberg method							
#Column "Input number" stands for the Count of the DE genes' entities directly associated with the listed Pathway ID							
#Column "Background number" stands for the total number of the DE genes' entities							
#Column " Enrichment ratio " equals "input number/ background number"							
Pathway ID	Term	P value	Enrichment score	Corrected P-Value	Input number	Background number	Enrichment ratio
dre00630	Glyoxylate and dicarboxylate metabolism	7.22E-05	4.14	0.00794257	7	33	0.212121212
dre00260	Glycine, serine and threonine metabolism	0.000445565	3.35	0.024506063	7	46	0.152173913
dre00983	Drug metabolism - other enzymes	0.006302348	2.2	0.21535897	5	40	0.125
dre00770	Pantothenate and CoA biosynthesis	0.007831235	2.11	0.21535897	3	13	0.230769231
dre00410	beta-Alanine metabolism	0.015678559	1.8	0.344928297	4	33	0.121212121
dre00250	Alanine, aspartate and glutamate metabolism	0.030063748	1.52	0.551168711	4	41	0.097560976
dre00051	Fructose and mannose metabolism	0.036952797	1.43	0.580686816	4	44	0.090909091
dre00380	Tryptophan metabolism	0.050289124	1.3	0.691475453	4	49	0.081632653
dre00910	Nitrogen metabolism	0.087953871	1.06	0.868544686	2	17	0.117647059
dre03030	DNA replication	0.097802446	1.01	0.868544686	3	39	0.076923077
dre01210	2-Oxocarboxylic acid metabolism	0.112949285	0.95	0.868544686	2	20	0.1
dre04630	Jak-STAT signaling pathway	0.121545103	0.92	0.868544686	6	122	0.049180328
dre01200	Carbon metabolism	0.124841806	0.9	0.868544686	6	123	0.048780488
dre00500	Starch and sucrose metabolism	0.143933723	0.84	0.868544686	3	47	0.063829787
dre00232	Caffeine metabolism	0.147836667	0.83	0.868544686	1	5	0.2
dre04510	Focal adhesion	0.151132393	0.82	0.868544686	10	251	0.039840637
dre00240	Pyrimidine metabolism	0.163959405	0.79	0.868544686	5	105	0.047619048
dre00524	Butirosin and neomycin biosynthesis	0.170263812	0.77	0.868544686	1	6	0.166666667
dre04012	ErbB signaling pathway	0.172629485	0.76	0.868544686	5	107	0.046728972
dre04068	FoxO signaling pathway	0.185689407	0.73	0.868544686	7	170	0.041176471
dre04920	Adipocytokine signaling pathway	0.201271728	0.7	0.868544686	4	84	0.047619048
dre04810	Regulation of actin cytoskeleton	0.206823314	0.68	0.868544686	10	271	0.036900369
dre04910	Insulin signaling pathway	0.207629531	0.68	0.868544686	7	176	0.039772727
dre00030	Pentose phosphate pathway	0.215111316	0.67	0.868544686	2	31	0.064516129
dre01230	Biosynthesis of amino acids	0.21777805	0.66	0.868544686	4	87	0.045977011
dre04622	RIG-I-like receptor signaling pathway	0.223096648	0.65	0.868544686	3	59	0.050847458
dre01100	Metabolic pathways	0.224395449	0.65	0.868544686	39	1269	0.030732861
dre00040	Pentose and glucuronate interconversions	0.22485227	0.65	0.868544686	2	32	0.0625

dre04146	Peroxisome	0.228979963	0.64	0.868544686	4	89	0.04494382
dre04141	Protein processing in endoplasmic reticulum	0.238297879	0.62	0.873758889	7	184	0.038043478
dre00310	Lysine degradation	0.26541854	0.58	0.932648901	3	65	0.046153846
dre04620	Toll-like receptor signaling pathway	0.310551922	0.51	0.932648901	4	103	0.038834951

Table S8. Pathway analysis results of genes with increased expression in *lyz-gfp* embryos exposed to Cu^{2+} at 68 hpf (hours post fertilization).

#Column "Pathway ID" stands for Pathway identifiers used in KEGG

#Column "Term" stands for the definition of the Pathway ID

#Column "P value" stands for the enrichment p-value of the Pathway ID as calculated by the Fisher's exact test

#Column "Enrichment Score" stands for the Enrichment Score value of the Pathway ID, which equals "(-log10(P-value))"

#Column "Corrected P-Value" stands for the false discovery rate of the Pathway ID as estimated by the Benjamini & Hochberg method

#Column "Input number" stands for the Count of the DE genes' entities directly associated with the listed Pathway ID

#Column "Background number" stands for the total number of the DE genes' entities

#Column " Enrichment ratio " equals "input number/ background number"

Pathway ID	Term	P value	Enrichment score	Corrected P-Value	Input number	Background number	Enrichment ratio
dre04744	Phototransduction	0.000445303	3.351344466	0.020740177	6	40	0.15
dre04145	Phagosome	0.000455828	3.341198948	0.020740177	12	164	0.073170732
dre04260	Cardiac muscle contraction	0.002005933	2.697683502	0.060846645	8	97	0.082474227
dre04540	Gap junction	0.009561394	2.019478799	0.217521706	8	128	0.0625
dre01230	Biosynthesis of amino acids	0.015604297	1.806755802	0.283998199	6	87	0.068965517
dre00430	Taurine and hypotaurine metabolism	0.022656964	1.644798285	0.343630621	2	9	0.222222222
dre04261	Adrenergic signaling in cardiomyocytes	0.037681101	1.423876415	0.489854314	9	199	0.045226131
dre00190	Oxidative phosphorylation	0.04773412	1.321171076	0.542975619	7	145	0.048275862
dre00250	Alanine, aspartate and glutamate metabolism	0.069748023	1.156468098	0.616864604	3	41	0.073170732
dre00790	Folate biosynthesis	0.075127092	1.124203419	0.616864604	2	19	0.105263158
dre00670	One carbon pool by folate	0.075127092	1.124203419	0.616864604	2	19	0.105263158
dre04520	Adherens junction	0.088347393	1.053806262	0.616864604	5	104	0.048076923
dre00010	Glycolysis / Gluconeogenesis	0.089210109	1.049585932	0.616864604	4	74	0.054054054
dre04310	Wnt signaling pathway	0.097132045	1.012637468	0.616864604	7	173	0.040462428
dre04142	Lysosome	0.108089435	0.966216754	0.616864604	6	144	0.041666667
dre00650	Butanoate metabolism	0.108459711	0.964731558	0.616864604	2	24	0.083333333
dre00760	Nicotinate and nicotinamide metabolism	0.144858365	0.83905642	0.764394965	2	29	0.068965517
dre00030	Pentose phosphate pathway	0.160048272	0.79574901	0.764394965	2	31	0.064516129
dre00062	Fatty acid elongation	0.175512864	0.755691047	0.764394965	2	33	0.060606061
dre00410	beta-Alanine metabolism	0.175512864	0.755691047	0.764394965	2	33	0.060606061
dre04350	TGF-beta signaling pathway	0.176398838	0.75350428	0.764394965	4	97	0.041237113
dre04916	Melanogenesis	0.195661027	0.708495671	0.779335809	5	137	0.03649635
dre00750	Vitamin B6 metabolism	0.196974985	0.705588925	0.779335809	1	9	0.111111111
dre00740	Riboflavin metabolism	0.214406787	0.668761472	0.780440704	1	10	0.1
dre00920	Sulfur metabolism	0.214406787	0.668761472	0.780440704	1	10	0.1
dre00051	Fructose and mannose metabolism	0.263372825	0.579429038	0.819204214	2	44	0.045454545
dre00770	Pantothenate and CoA biosynthesis	0.264472323	0.577619769	0.819204214	1	13	0.076923077
dre04512	ECM-receptor interaction	0.268653143	0.570808075	0.819204214	3	80	0.0375
dre00260	Glycine, serine and threonine	0.279534303	0.55356489	0.819204214	2	46	0.043478261

	metabolism						
dre04010	MAPK signaling pathway	0.285939945	0.543725171	0.819204214	9	320	0.028125
dre04060	Cytokine-cytokine receptor interaction	0.2922711	0.534214126	0.819204214	5	161	0.031055901
dre04621	NOD-like receptor signaling pathway	0.295670211	0.529192429	0.819204214	2	48	0.041666667

Fig.S1. The growth curve of *A. hydrophila* used in this study. Three biological replicates were performed. Date are presented as mean ± SD.

Fig.S2. Distribution of GFP positive cells in larvae after *A. hydrophila* infection in an immersion only manner (**A, B, C**), FACS (flow cytometry) plots (**D1-D8)** and percentages of *lyz* promotor driven GFP positive cells in copper stressed and control larvae with and without *A. hydrophila* infection (**E**). The transgenic zebrafish larvae of *coro1a*-GFP, *lyz*-GFP, were infected with *A. hydrophila via* immersion at 68 hpf. 3 biological replicates were performed. ANOVA-post hoc Turkey's Test. Date are presented as mean ± SD. ***, $P < 0.001$; **, $P < 0.01$; *, $P < 0.05$ and ns, no significance.

Fig.S3. The recruitment of GFP positive *coro1a* macrophages (**A**), *mpx* neutrophils (**B**), and *lyz* neutrophils (**C**) in the fixed domain (red boxes) around injury locus was analyzed at 0, 2, 4, and 6 hours post *A. hydrophila* infection.

Fig.S4. Error rate distribution along reads for samples of *coro1a* control-68hpf, *coro1a* Cu^{2+}-68hpf, *mpx* control-68hpf, *mpx* Cu^{2+}-68hpf, *lyz* control-68hpf, *lyz* Cu^{2+}-68hpf respectively.

Fig.S5. Bases content along reads for sample of *coro1a* control-68hpf, *coro1a* Cu^{2+}-68hpf, *mpx* control-68hpf, *mpx* Cu^{2+}-68hpf, *lyz* control-68hpf, *lyz* Cu^{2+}-68hpf respectively.

Fig.S6. Classification of raw reads for sample of *coro1a* control-68hpf, *coro1a* Cu^{2+}-68hpf, *mpx* control-68hpf, *mpx* Cu^{2+}-68hpf, *lyz* control-68hpf, *lyz* Cu^{2+}-68hpf respectively.

Fig.S7. FPKM density distribution for sample of *coro1a* control-68hpf, *coro1a* Cu^{2+}-68hpf, *mpx* control-68hpf, *mpx* Cu^{2+}-68hpf, *lyz* control-68hpf, *lyz* Cu^{2+}-68hpf respectively.

Fig.S8. Pearson correlation between samples of *coro1a* control-68hpf *vs coro1a* Cu^{2+}-68hpf, *mpx* control-68hpf *vs mpx* Cu^{2+}-68hpf, and *lyz* control-68hpf *vs lyz*

Cu^{2+}-68hpf respectively.

Fig.S9. DEGs were screened in sample of *coro1a* Cu^{2+}-68hpf after compared to *coro1a* control-68hpf at the adjusted $P<0.05$.

Fig.S10. DEGs were screened in sample of *mpx* Cu^{2+}-68hpf after compared to *mpx* control-68hpf at the adjusted $P<0.05$.

Fig.S11. DEGs were screened in sample of *lyz* Cu^{2+}-68hpf after compared to *lyz* control-68hpf at the adjusted $P<0.05$.

Fig.S12. The clustering analysis of genes related to proteasome and lysosome in GFP-labeled cells of copper-treated transgenic larvae (**A-F**) and expression of the representative proteasome and lysosome genes in macrophages or neutrophils *via* cell direct qRT-PCR detection (**G**). 3 biological replicates were performed. ANOVA-post hoc Turkey's Test. Date are presented as mean ± SD. ***, $P < 0.001$; **, $P < 0.01$; *, $P < 0.05$ and ns, no significance.

Fig.S13. The clustering analysis of genes related to apoptosis in *coro1a* driven GFP-labeled cells of copper-treated transgenic larvae.

Fig.S14. Percentage of PI (**A1**, **A2**) labelled macrophages and neutrophils in copper-stressed larvae. FACS plots (**B2-B7**) for Annexin-V labelled cells in copper-stressed non-GFP cells in *coro1a* (**B2**, **B3**), *mpx* (**B4**, **B5**), and *lyz* transgenic larvae (**B6**, **B7**), the green box indicting non-GFP cells used for Annexin-V PE staining analysis in this study (B1), the blue box indicated the selected non GFP cells labeled by Annexin V-PE. Percentage of annexin V-PE labelled non-GFP cells in copper-stressed larvae (**C**). 3 biological replicates were performed. ANOVA-post hoc Turkey's Test. Date are presented as mean ± SD. ***, $P < 0.001$; **, $P < 0.01$; *, $P < 0.05$ and ns, no significance.

Fig.S15. The clustering analysis of genes related to oxidative phosphorylation in GFP-labeled cells of copper-treated transgenic larvae (**A-C**) and expression of the representative oxidative phosphorylation genes in macrophages or neutrophils *via* cell

direct qRT-PCR detection (**D**). 3 biological replicates were performed. ANOVA-post hoc Turkey's Test. Date are presented as mean ± SD. ***, $P < 0.001$; **, $P < 0.01$; *, $P < 0.05$ and ns, no significance.

Fig.S16. FACS plots (**A1-A4 and A6-A9**) and relative ROS fluorescence for neutrophils in copper stressed *mpx* (**A5**) and *lyz* promotor driven GFP transgenic larvae (**A6**). The red boxed indicated GFP and ROS double positive cells. 3 biological replicates were performed. ANOVA-post hoc Turkey's Test. Date are presented as mean ± SD. ***, $P < 0.001$; **, $P < 0.01$; *, $P < 0.05$ and ns, no significance.

Fig.S17. Expression of different caspase genes in copper stressed neutrophils in *mpx* promotor driven GFP transgenic larvae before and after *A. hydrophila* infection (**A1**), and expression of mROS-mediated apoptosis genes in the cells before and after *A. hydrophila* infection (**A2**). 3 biological replicates were performed. ANOVA-post hoc Turkey's Test. Date are presented as mean ± SD. ***, $P < 0.001$; **, $P < 0.01$; *, $P < 0.05$ and ns, no significance.

Fig.S18. Expression of different caspase genes in copper stressed neutrophils in *lyz* promotor driven GFP transgenic larvae before and after *A. hydrophila* infection (**A1**), and expression of mROS-mediated apoptosis genes in the cells before and after *A. hydrophila* infection (**A2**). 3 biological replicates were performed. ANOVA-post hoc Turkey's Test. Date are presented as mean ± SD. ***, $P < 0.001$; **, $P < 0.01$; *, $P < 0.05$ and ns, no significance.

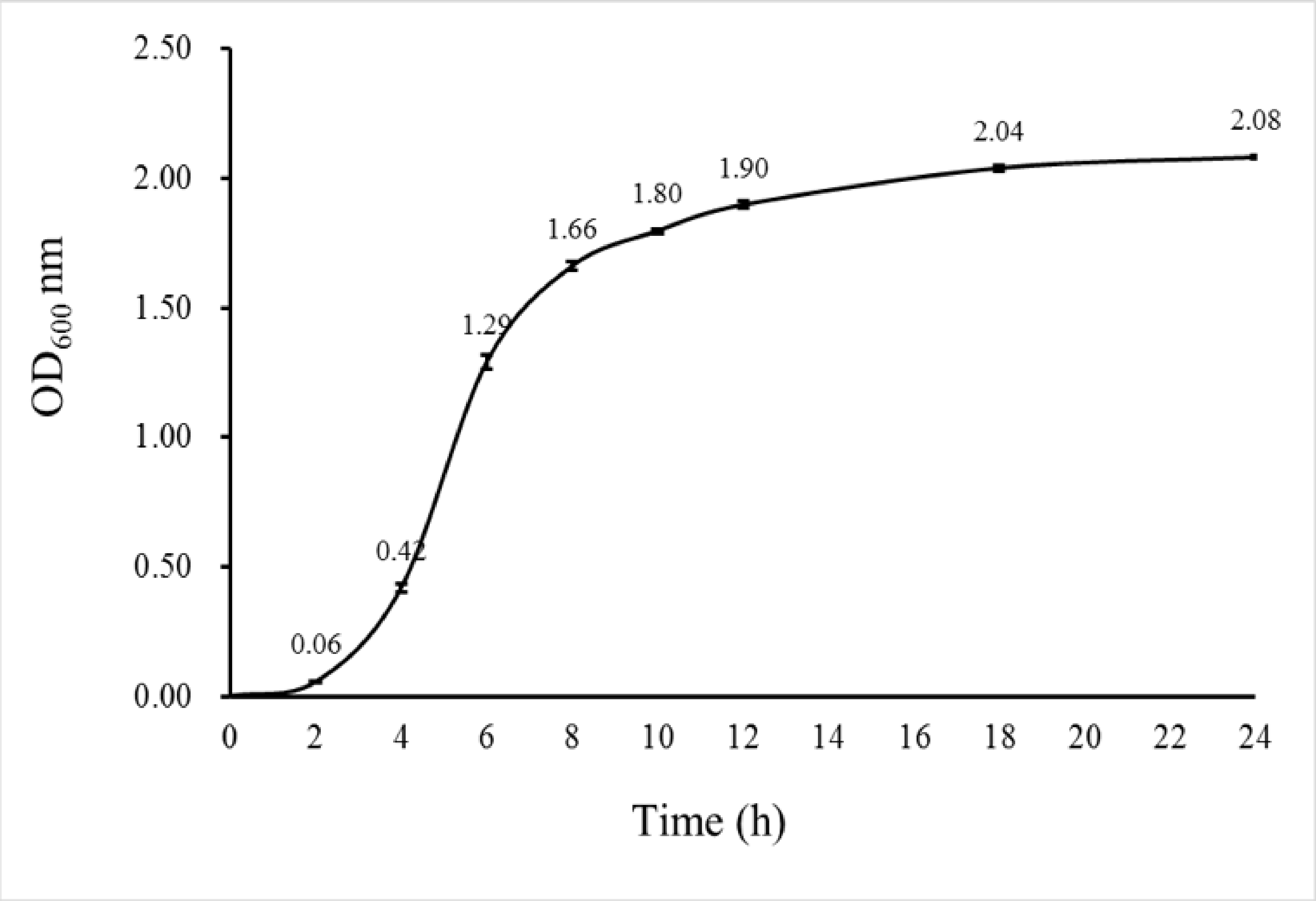

2.50
2.00
1.50
1.00
0.50
0.00
OD600 nm
0.06
0.42
1.29
1.66
1.80
1.90
2.04
2.08
0
2
4
6
8
10
12
14
16
18
20
22
24
Time (h)

Fig.S1

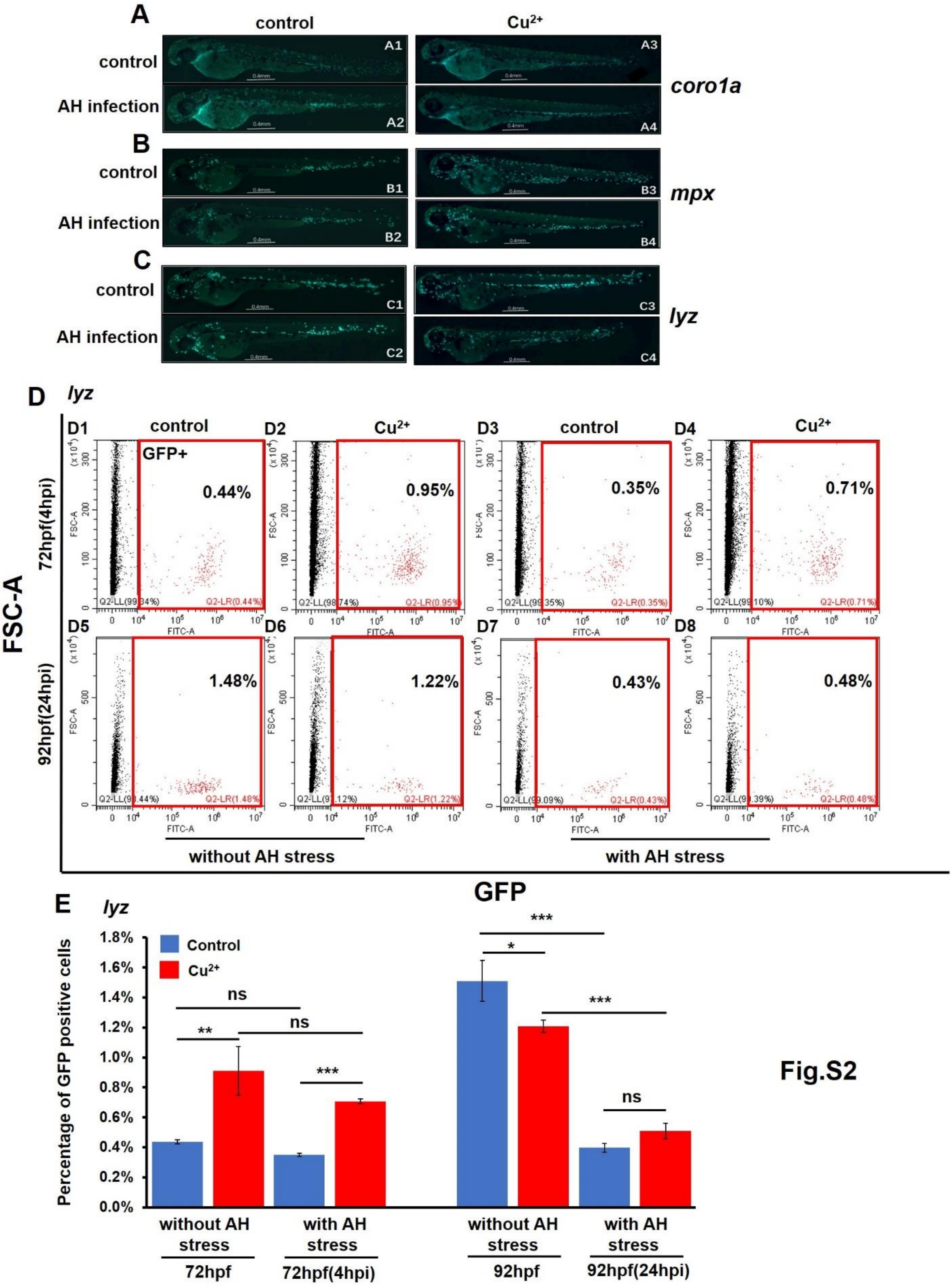
A
control
Cu2+
control
AH infection
coro1a
B
control
AH infection
mpx
C
control
AH infection
lyz
D lyz
FSC-A
72hpf(4hpi)
92hpf(24hpi)
D1 control
GFP+
0.44%
D2 Cu2+
0.95%
D3 control
0.35%
D4 Cu2+
0.71%
D5
1.48%
D6
1.22%
D7
0.43%
D8
0.48%
FITC-A
without AH stress
with AH stress
GFP
E lyz
Percentage of GFP positive cells
Control
Cu2+
ns
**
ns
***
***
*
***
ns
without AH stress
72hpf
with AH stress
72hpf(4hpi)
without AH stress
92hpf
with AH stress
92hpf(24hpi)

Fig.S2

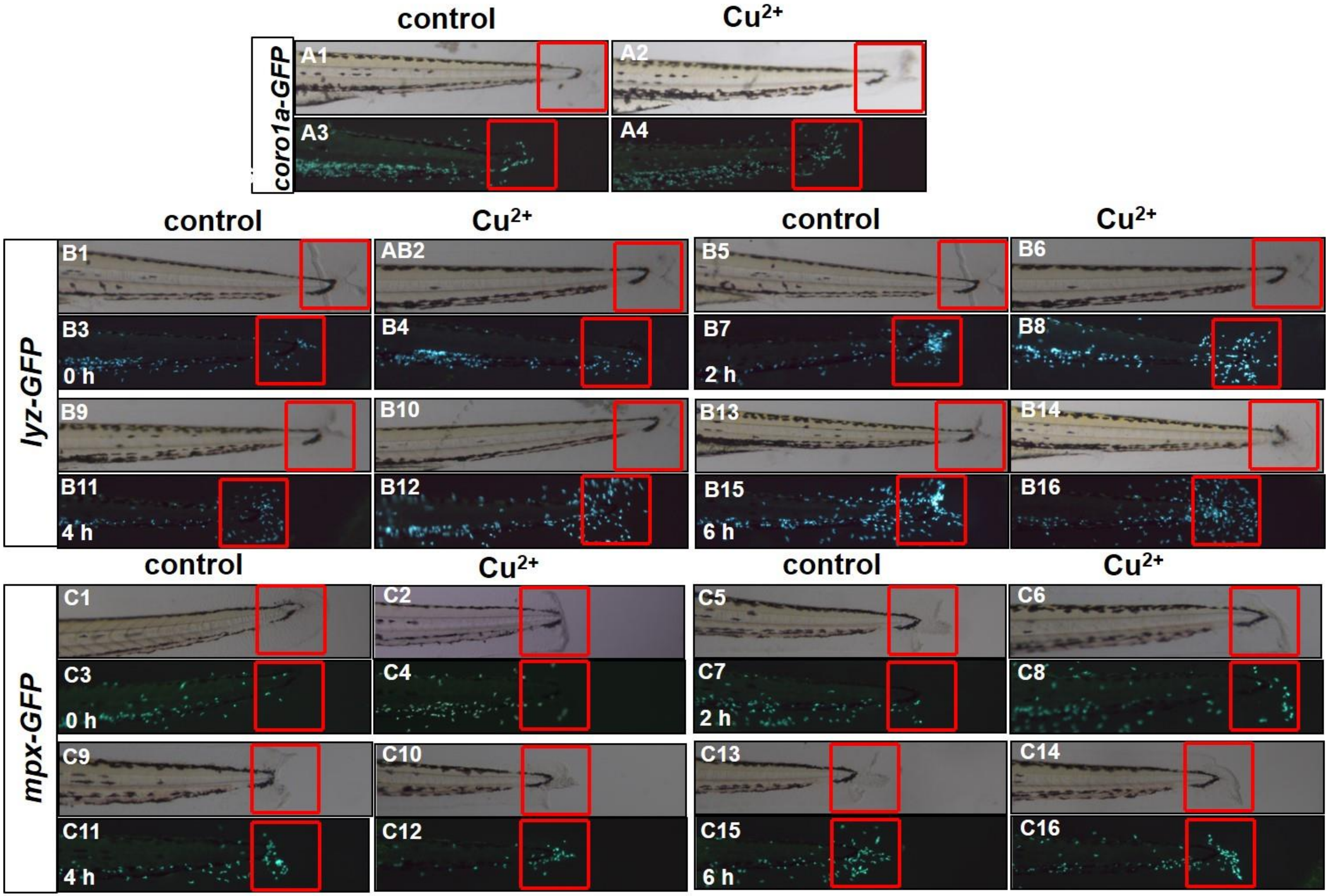
control
Cu2+
coro1a-GFP
A1
A2
A3
A4
control
Cu2+
control
Cu2+
lyz-GFP
B1
AB2
B5
B6
B3
0 h
B4
B7
2 h
B8
B9
B10
B13
B14
B11
4 h
B12
B15
6 h
B16
control
Cu2+
control
Cu2+
mpx-GFP
C1
C2
C5
C6
C3
0 h
C4
C7
2 h
C8
C9
C10
C13
C14
C11
4 h
C12
C15
6 h
C16

Fig.S3

Error rate distribution along reads

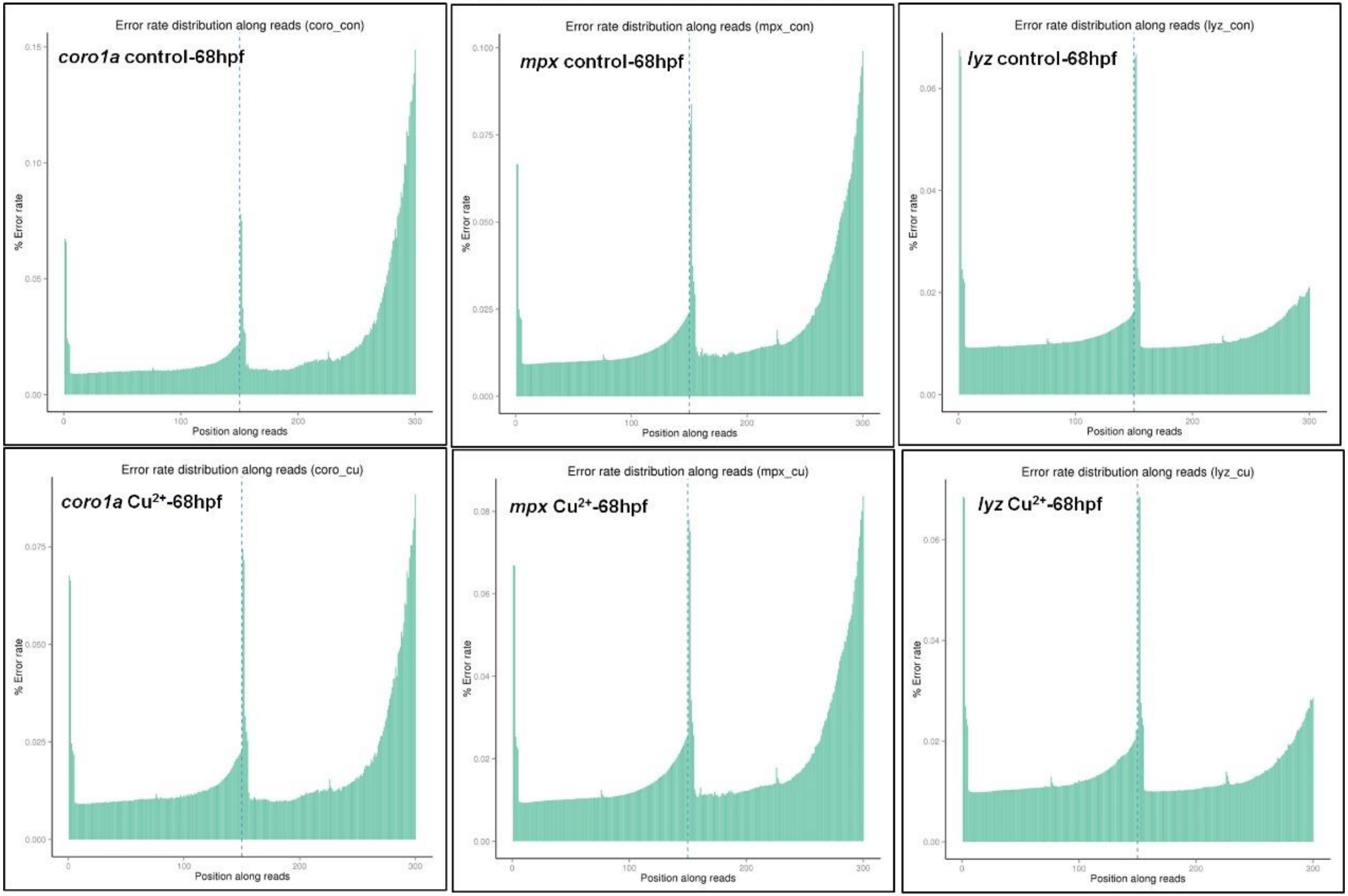

Fig.S4

bases content along reads

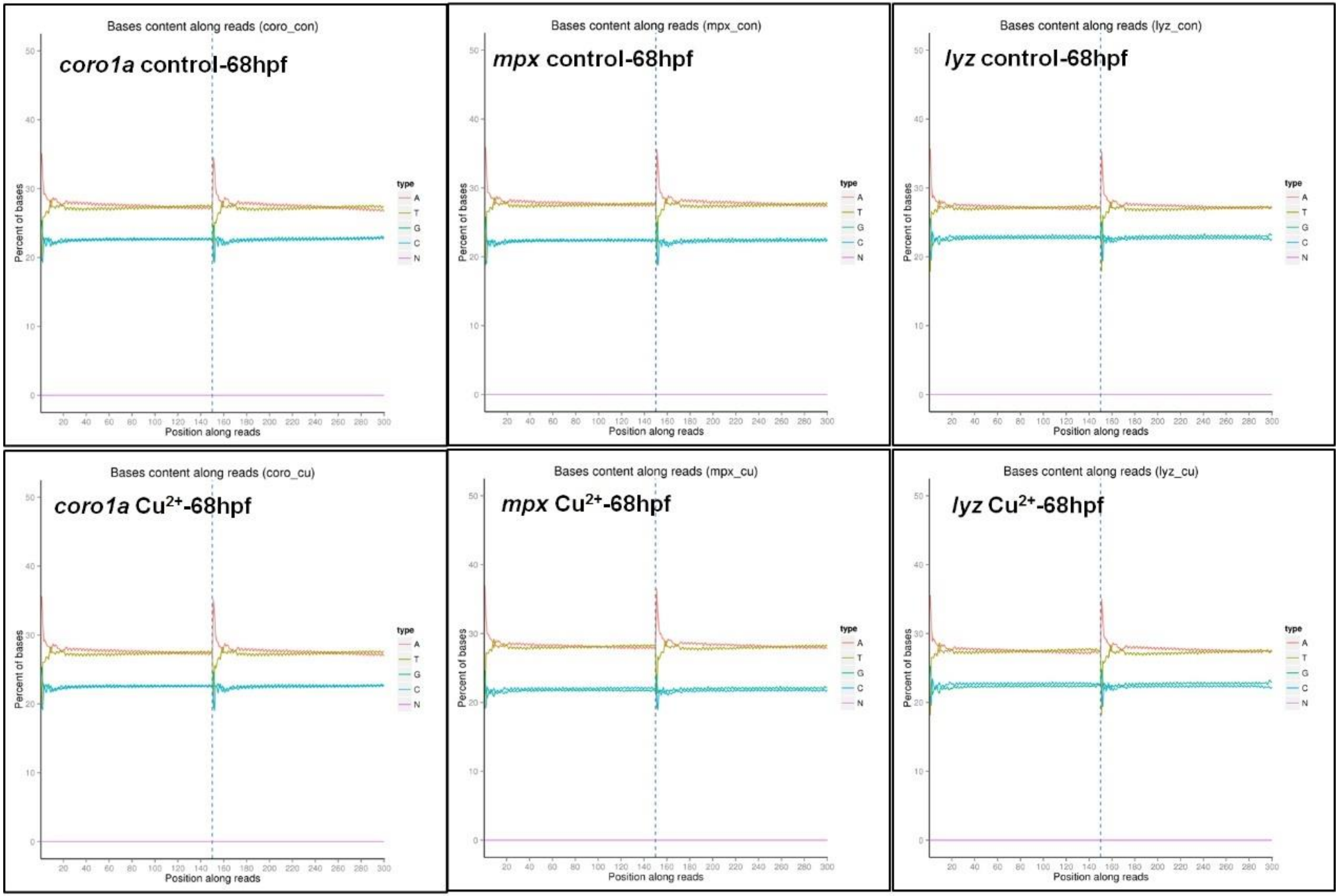

Fig.S5

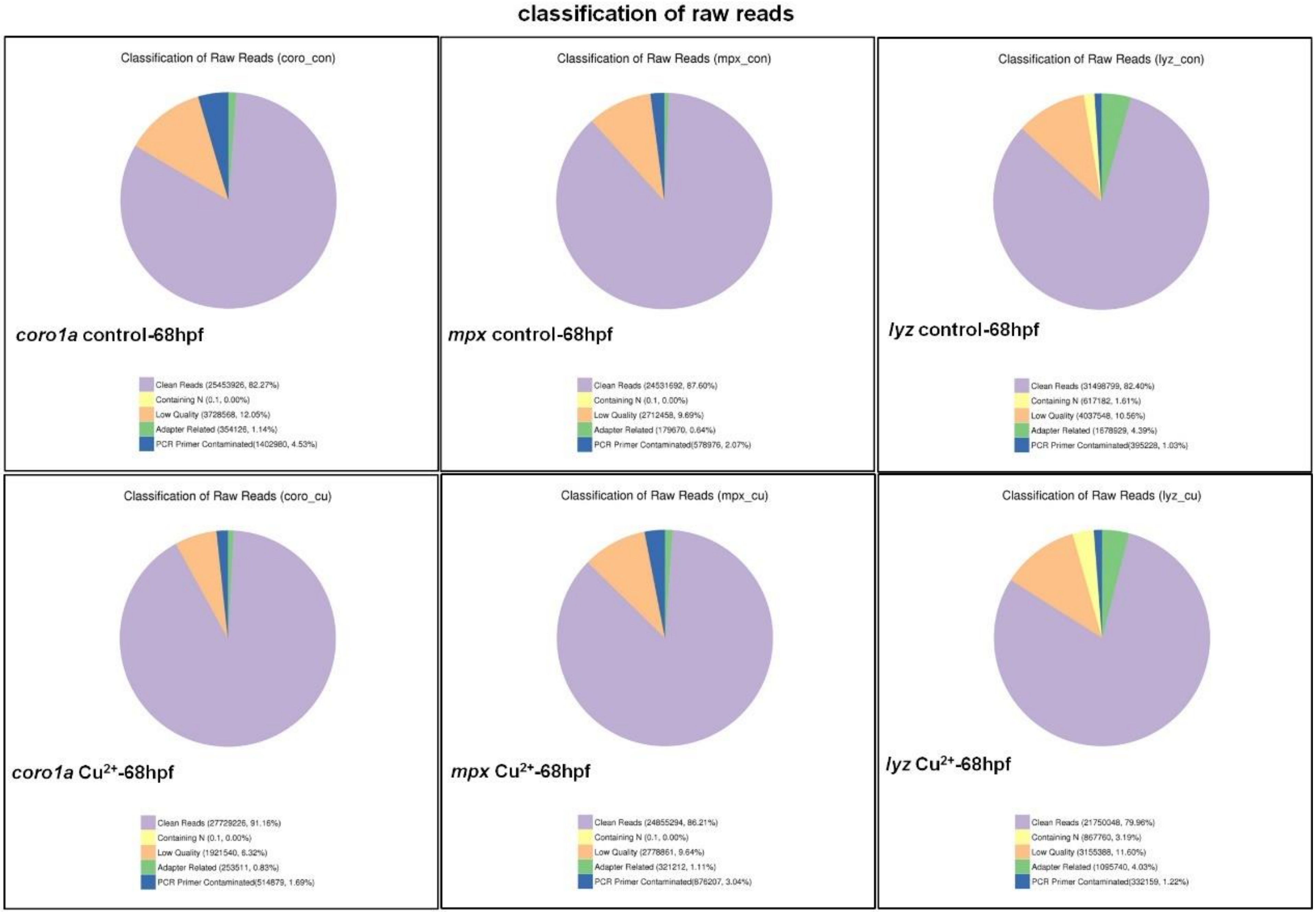
classification of raw reads
Classification of Raw Reads (coro_con)
coro1a control-68hpf
Clean Reads (25453926, 82.27%)
Containing N (0.1, 0.00%)
Low Quality (3728568, 12.05%)
Adapter Related (354126, 1.14%)
PCR Primer Contaminated(1402980, 4.53%)
Classification of Raw Reads (mpx_con)
mpx control-68hpf
Clean Reads (24531692, 87.60%)
Containing N (0.1, 0.00%)
Low Quality (2712458, 9.69%)
Adapter Related (179670, 0.64%)
PCR Primer Contaminated(578976, 2.07%)
Classification of Raw Reads (lyz_con)
lyz control-68hpf
Clean Reads (31498799, 82.40%)
Containing N (617182, 1.61%)
Low Quality (4037548, 10.56%)
Adapter Related (1678929, 4.39%)
PCR Primer Contaminated(395228, 1.03%)
Classification of Raw Reads (coro_cu)
coro1a Cu2+-68hpf
Clean Reads (27729226, 91.16%)
Containing N (0.1, 0.00%)
Low Quality (1921540, 6.32%)
Adapter Related (253511, 0.83%)
PCR Primer Contaminated(514879, 1.69%)
Classification of Raw Reads (mpx_cu)
mpx Cu2+-68hpf
Clean Reads (24855294, 86.21%)
Containing N (0.1, 0.00%)
Low Quality (2778861, 9.64%)
Adapter Related (321212, 1.11%)
PCR Primer Contaminated(876207, 3.04%)
Classification of Raw Reads (lyz_cu)
lyz Cu2+-68hpf
Clean Reads (21750048, 79.96%)
Containing N (867760, 3.19%)
Low Quality (3155388, 11.60%)
Adapter Related (1095740, 4.03%)
PCR Primer Contaminated(332159, 1.22%)

Fig.S6

FPKM density distribution

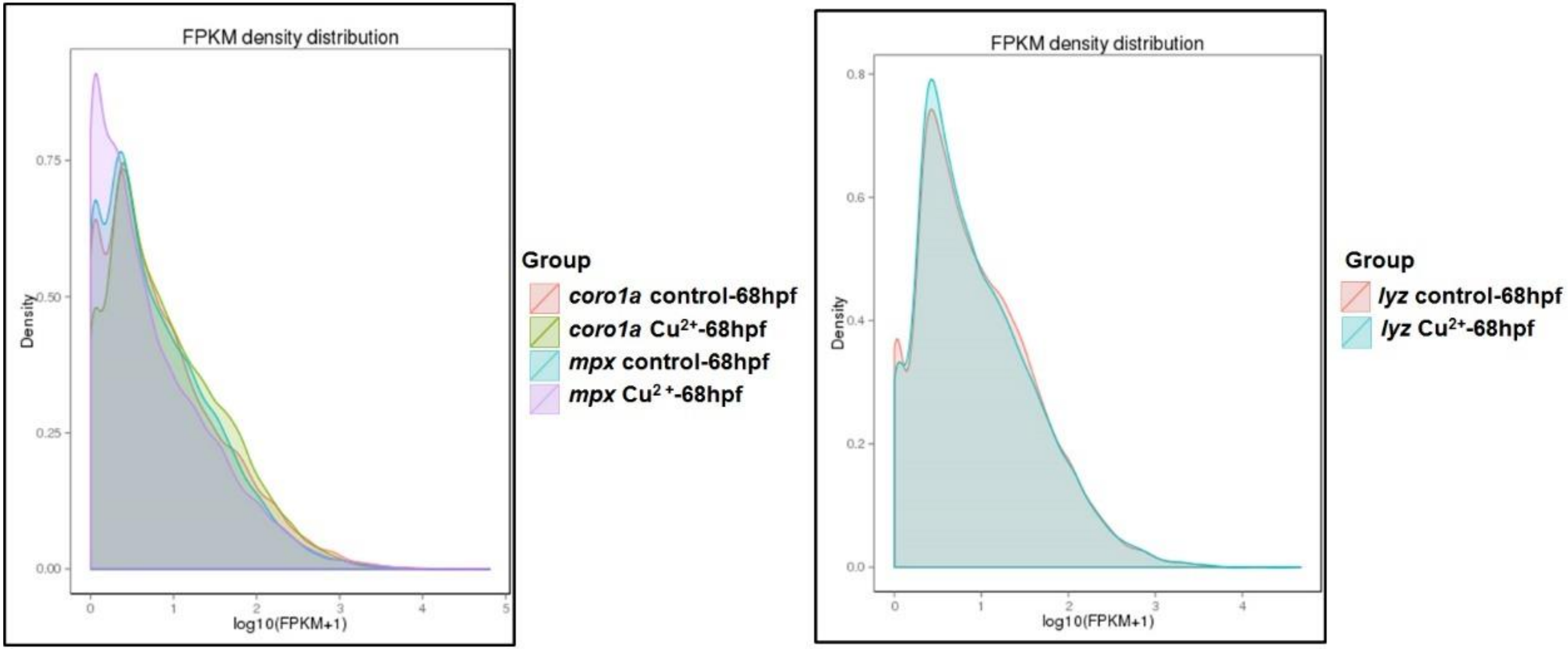

Fig.S7

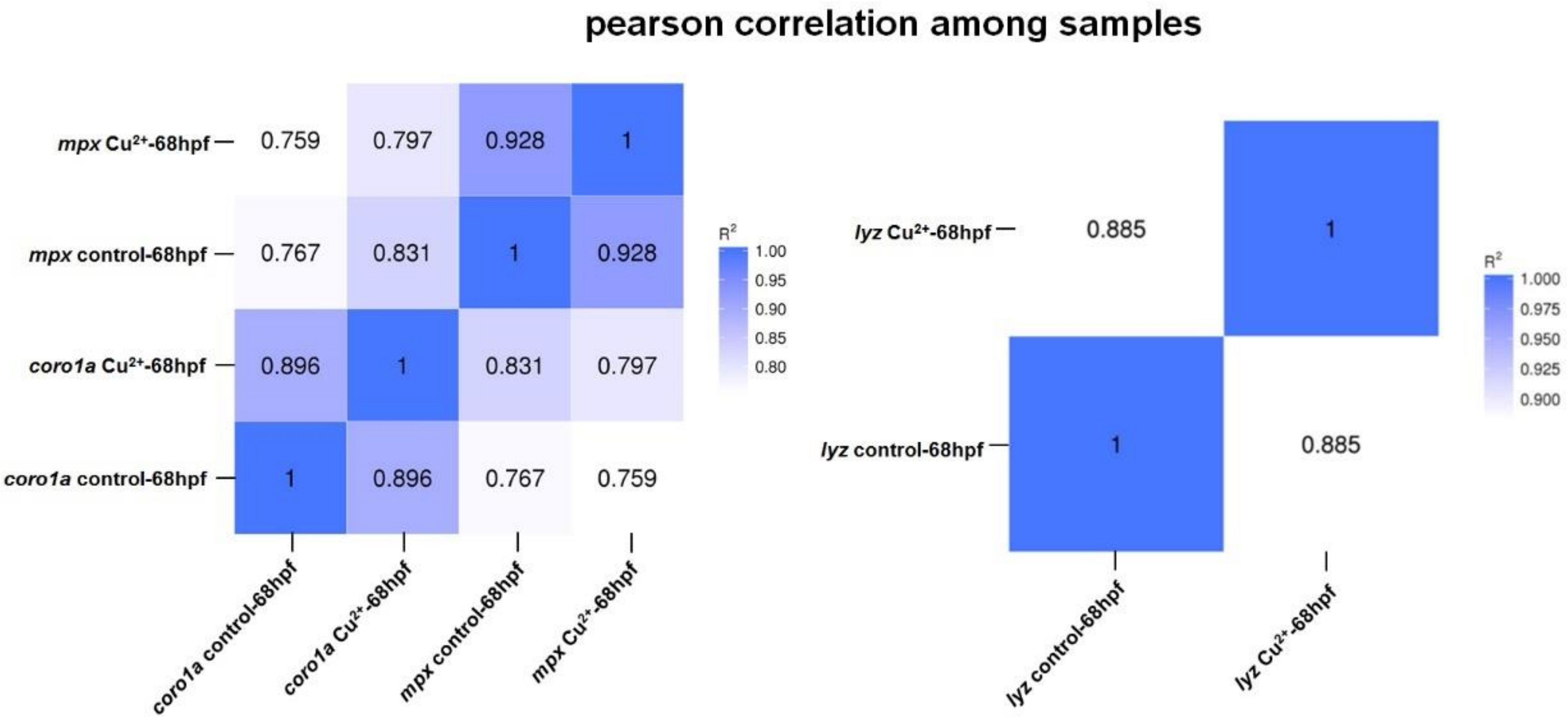

pearson correlation among samples
mpx Cu2+-68hpf
mpx control-68hpf
coro1a Cu2+-68hpf
coro1a control-68hpf
0.759
0.797
0.928
1
0.767
0.831
1
0.928
0.896
1
0.831
0.797
1
0.896
0.767
0.759
coro1a control-68hpf
coro1a Cu2+-68hpf
mpx control-68hpf
mpx Cu2+-68hpf
R2
1.00
0.95
0.90
0.85
0.80
lyz Cu2+-68hpf
lyz control-68hpf
0.885
1
1
0.885
lyz control-68hpf
lyz Cu2+-68hpf
R2
1.000
0.975
0.950
0.925
0.900

Fig.S8

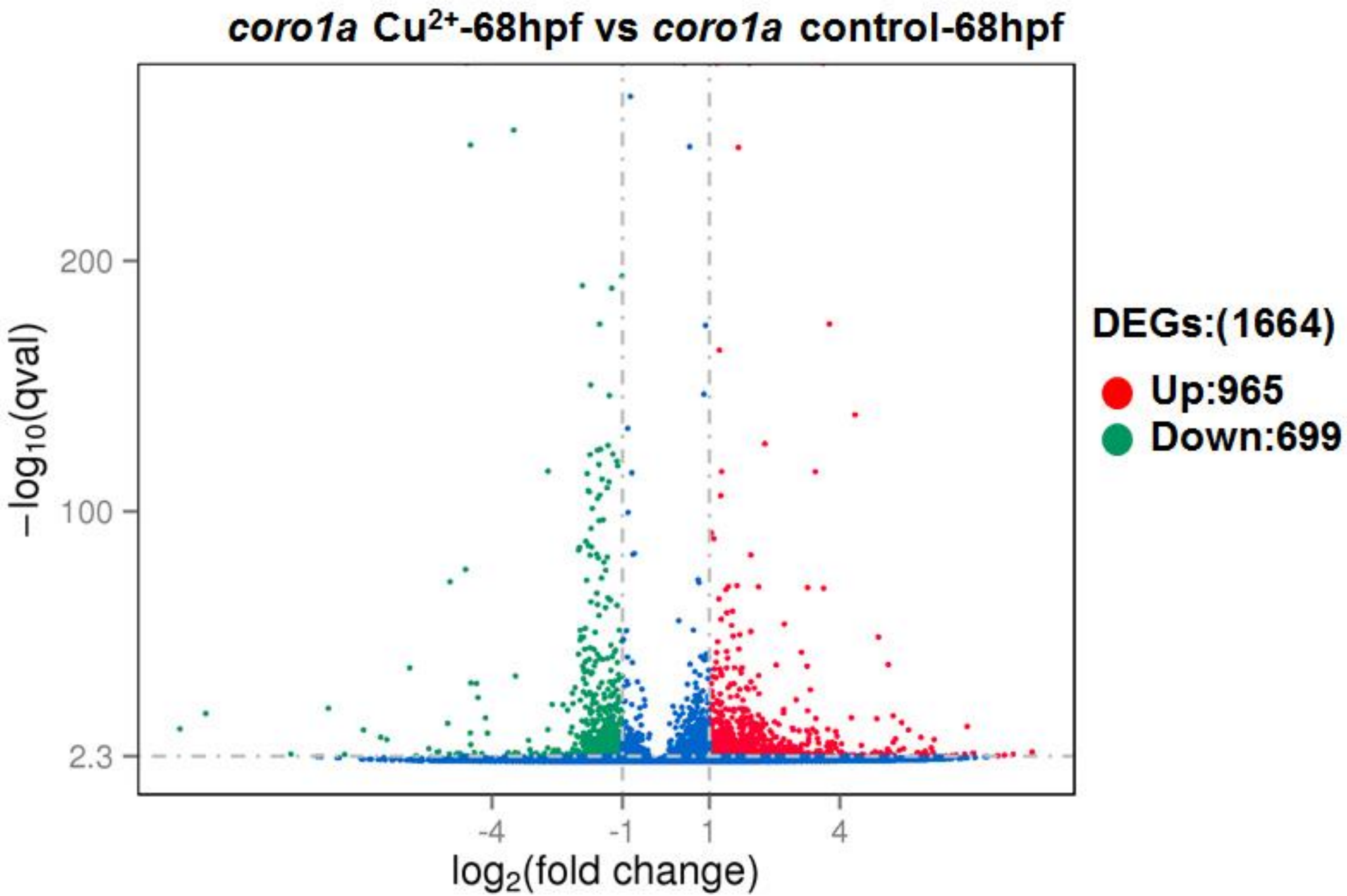
coro1a Cu2+-68hpf vs *coro1a* control-68hpf
200
100
2.3
−log10(qval)
-4
-1
1
4
log2(fold change)
DEGs:(1664)
Up:965
Down:699

Fig.S9

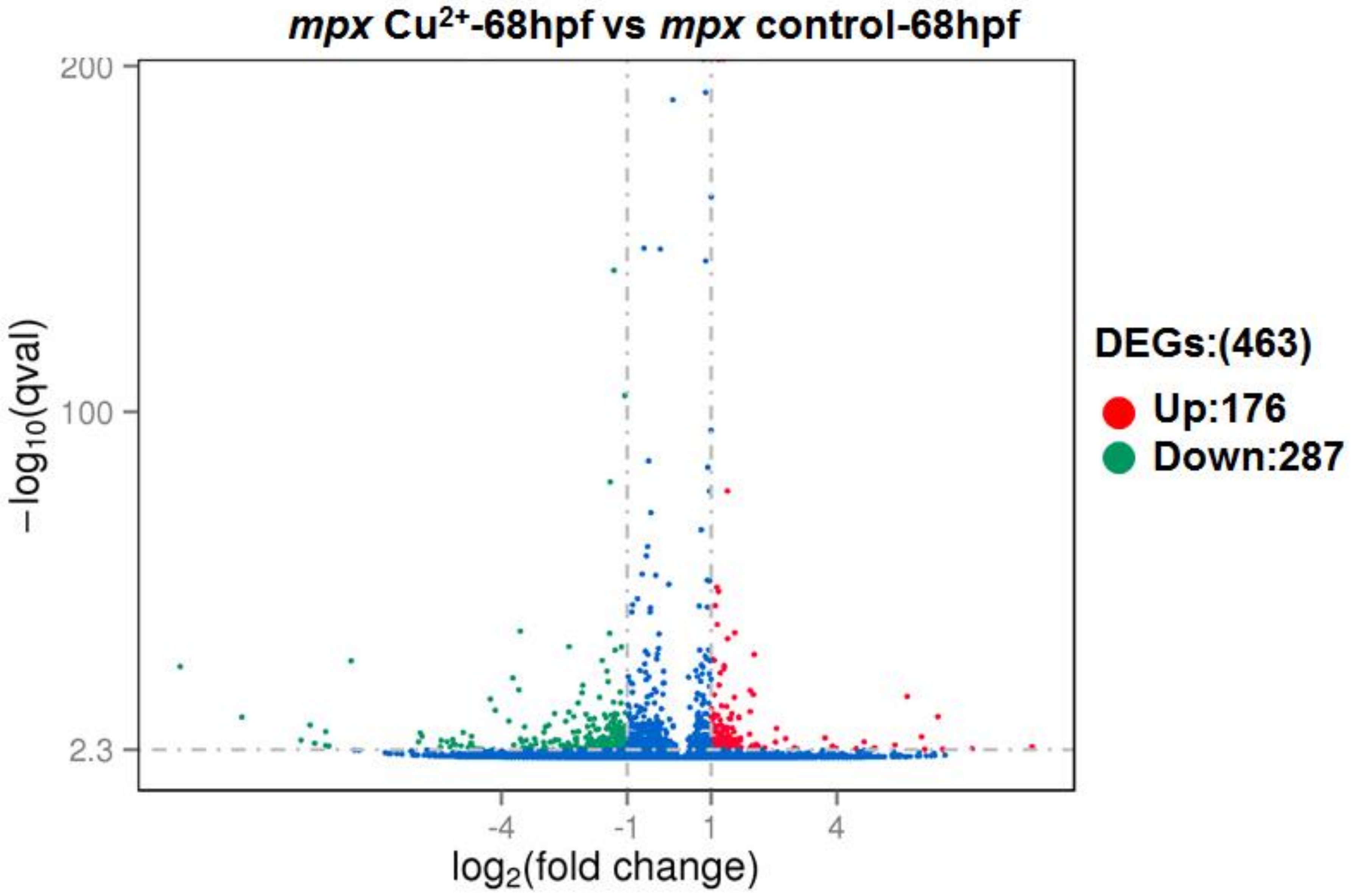
mpx Cu2+-68hpf vs mpx control-68hpf
200
100
2.3
−log10(qval)
-4
-1
1
4
log2(fold change)
DEGs:(463)
Up:176
Down:287

Fig.S10

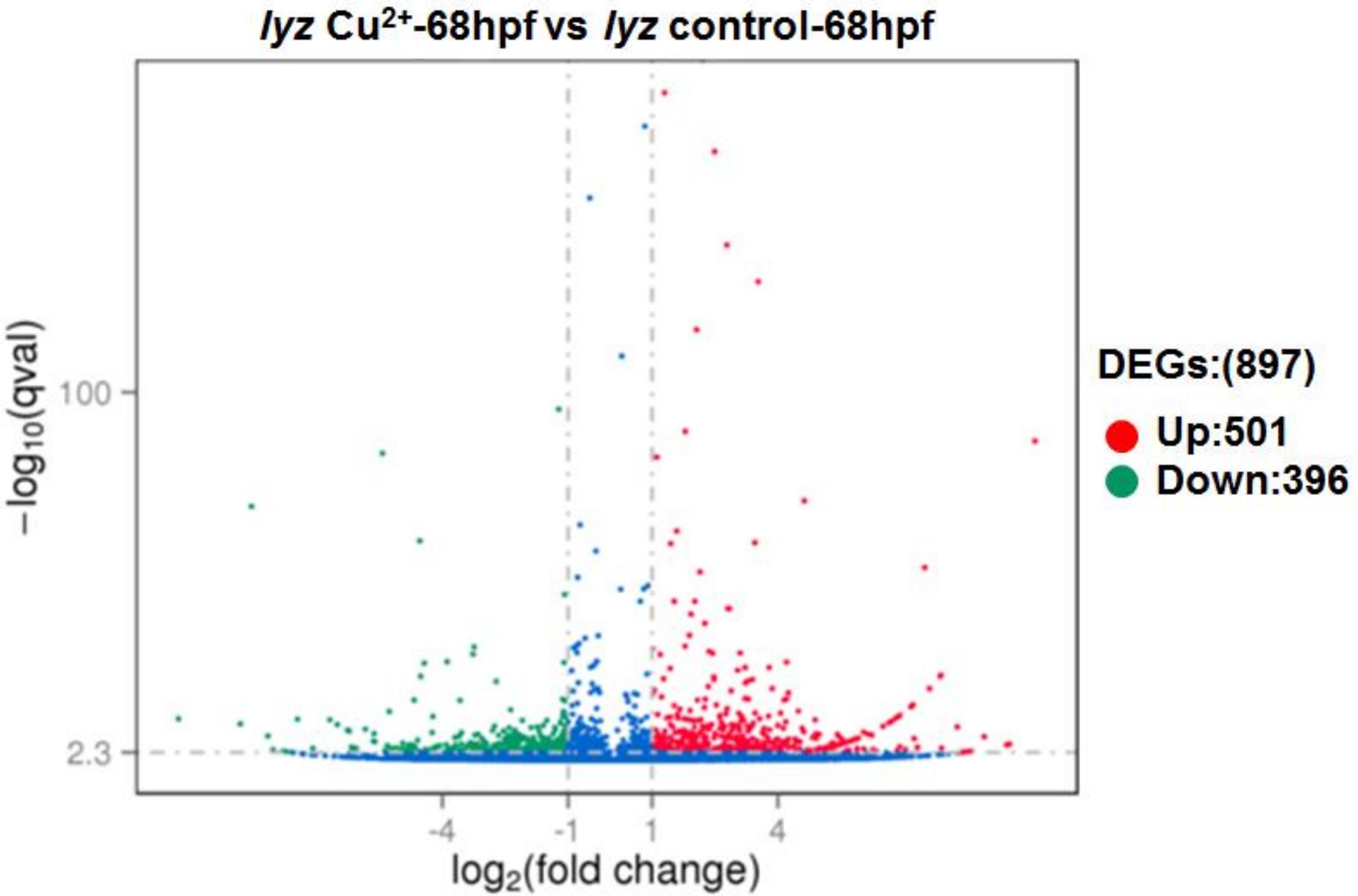

lyz Cu2+-68hpf vs *lyz* control-68hpf
−log10(qval)
100
2.3
-4
-1
1
4
log2(fold change)
DEGs:(897)
Up:501
Down:396

Fig.S11

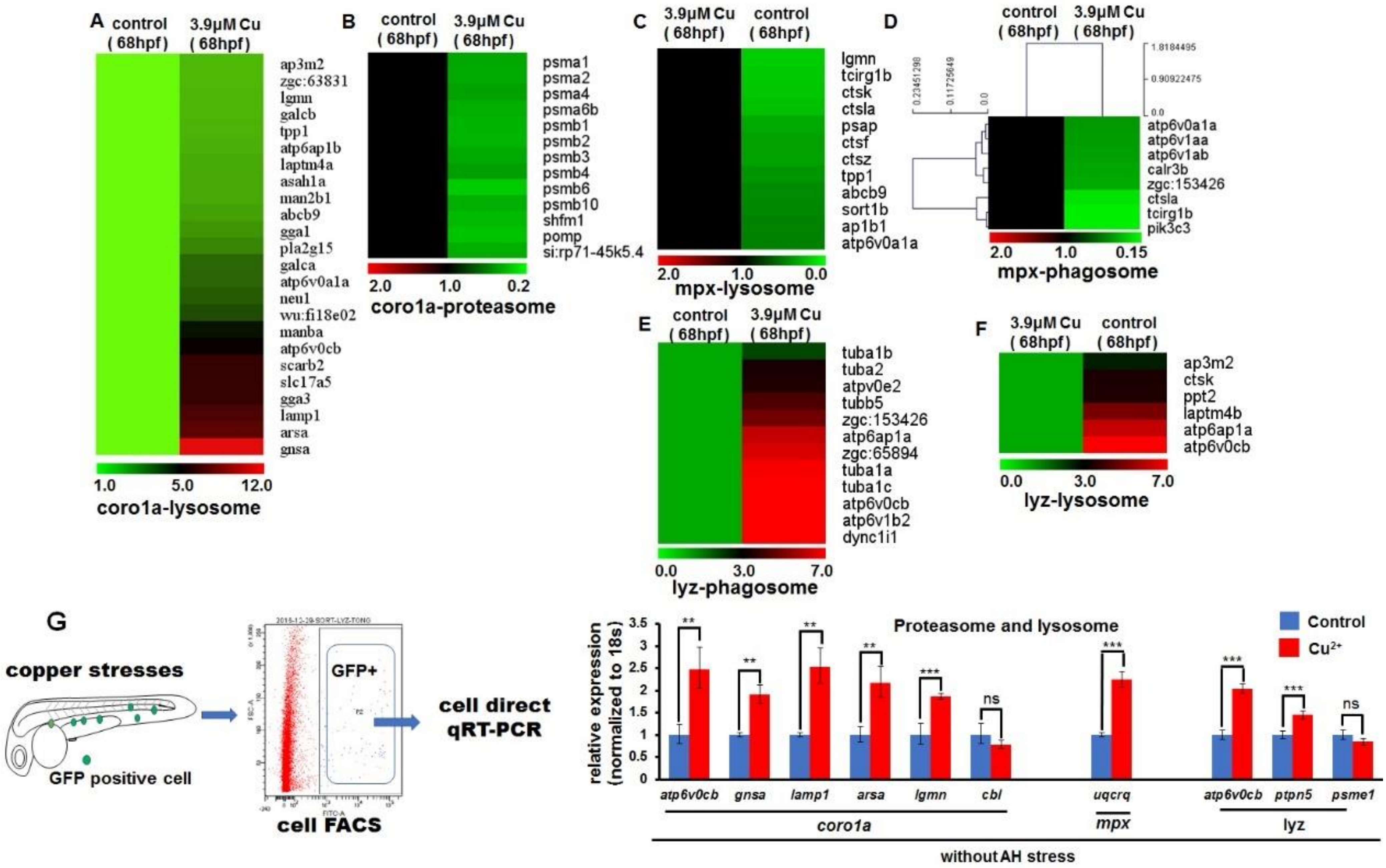

A
control (68hpf)
3.9μM Cu (68hpf)
ap3m2
zgc:63831
lgmn
galcb
tpp1
atp6ap1b
laptm4a
asah1a
man2b1
abcb9
gga1
pla2g15
galca
atp6v0a1a
neu1
wu:fi18e02
manba
atp6v0cb
scarb2
slc17a5
gga3
lamp1
arsa
gnsa
1.0 5.0 12.0
coro1a-lysosome
B
control (68hpf)
3.9μM Cu (68hpf)
psma1
psma2
psma4
psma6b
psmb1
psmb2
psmb3
psmb4
psmb6
psmb10
shfm1
pomp
si:rp71-45k5.4
2.0 1.0 0.2
coro1a-proteasome
C
3.9μM Cu (68hpf)
control (68hpf)
lgmn
tcirg1b
ctsk
ctsla
psap
ctsf
ctsz
tpp1
abcb9
sort1b
ap1b1
atp6v0a1a
2.0 1.0 0.0
mpx-lysosome
D
control (68hpf)
3.9μM Cu (68hpf)
1.8184495
0.90922475
0.0
0.23451298
0.11725649
0.0
atp6v0a1a
atp6v1aa
atp6v1ab
calr3b
zgc:153426
ctsla
tcirg1b
pik3c3
2.0 1.0 0.15
mpx-phagosome
E
control (68hpf)
3.9μM Cu (68hpf)
tuba1b
tuba2
atpv0e2
tubb5
zgc:153426
atp6ap1a
zgc:65894
tuba1a
tuba1c
atp6v0cb
atp6v1b2
dync1i1
0.0 3.0 7.0
lyz-phagosome
F
3.9μM Cu (68hpf)
control (68hpf)
ap3m2
ctsk
ppt2
laptm4b
atp6ap1a
atp6v0cb
0.0 3.0 7.0
lyz-lysosome
G
copper stresses
GFP positive cell
GFP+
cell FACS
cell direct qRT-PCR
Proteasome and lysosome
Control
Cu2+
relative expression (normalized to 18s)
3.5
3
2.5
2
1.5
1
0.5
0
**
**
**
**
***
ns
***
***
***
ns
atp6v0cb
gnsa
lamp1
arsa
lgmn
cbl
uqcrq
atp6v0cb
ptpn5
psme1
coro1a
mpx
lyz
without AH stress

Fig.S12

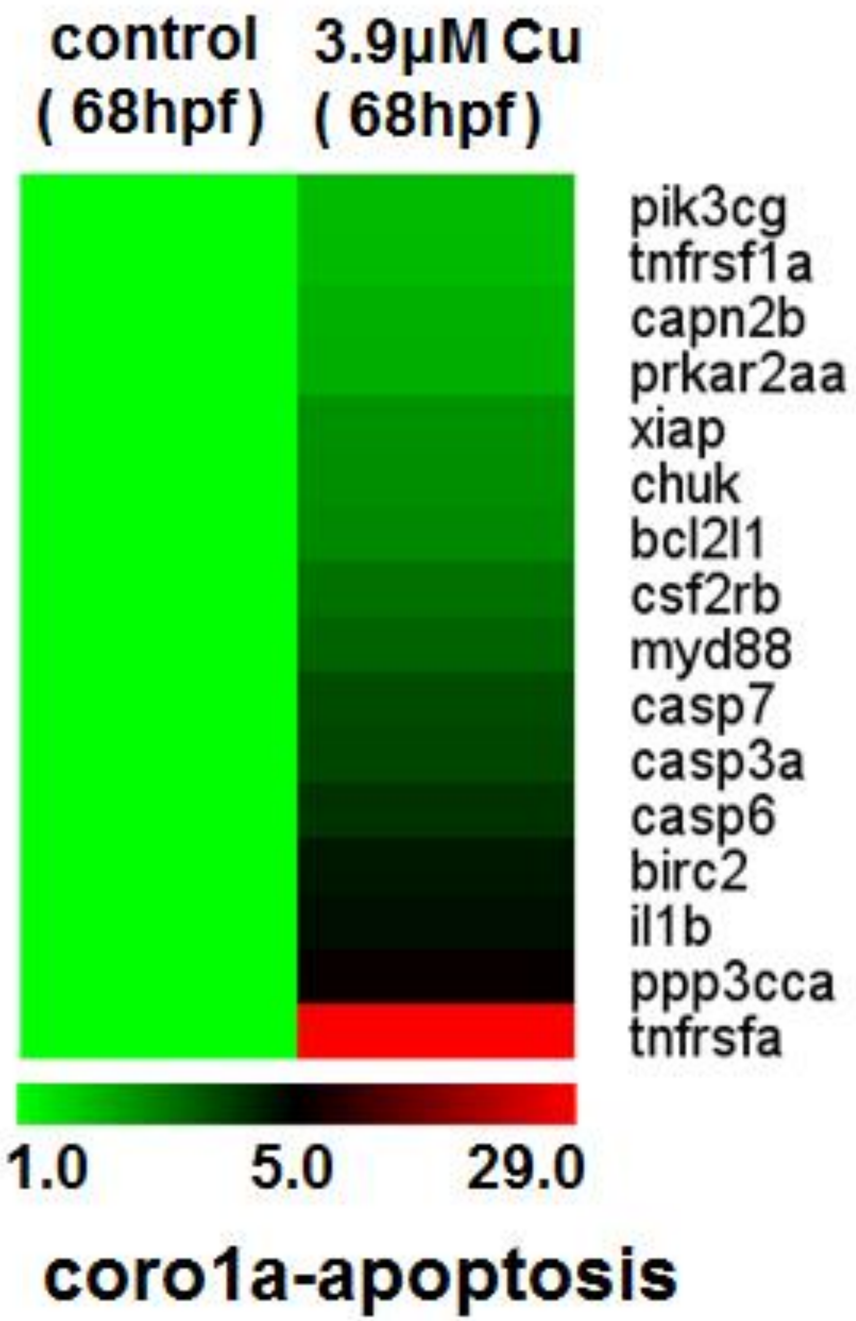

control
(68hpf)
3.9μM Cu
(68hpf)
pik3cg
tnfrsf1a
capn2b
prkar2aa
xiap
chuk
bcl2l1
csf2rb
myd88
casp7
casp3a
casp6
birc2
il1b
ppp3cca
tnfrsfa
1.0
5.0
29.0
coro1a-apoptosis

Fig.S13

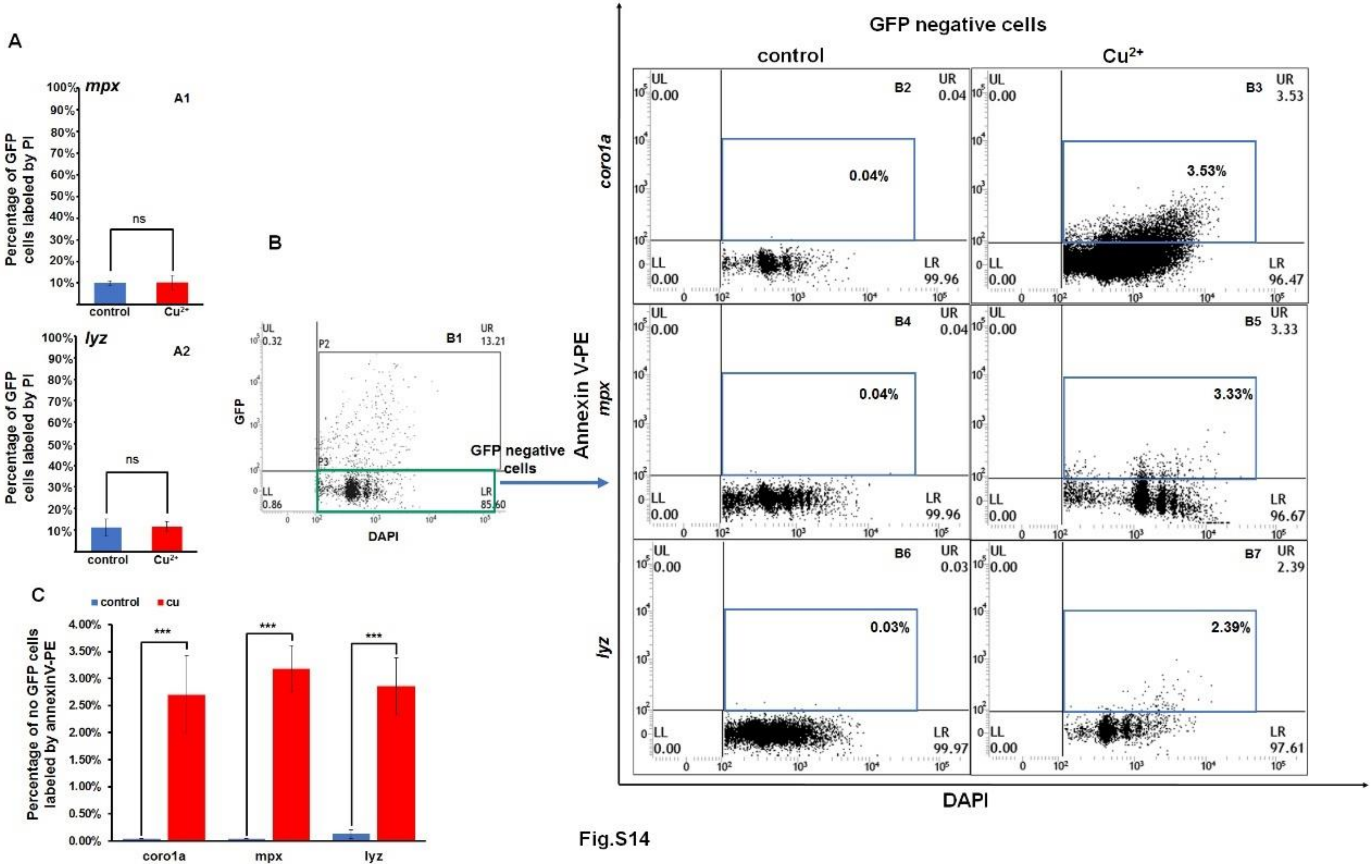
A
mpx
A1
Percentage of GFP cells labeled by PI
ns
control
Cu²⁺
lyz
A2
B
B1
GFP
DAPI
GFP negative cells
GFP negative cells
control
Cu²⁺
coro1a
mpx
lyz
Annexin V-PE
DAPI
B2
B3
B4
B5
B6
B7
0.04%
3.53%
0.04%
3.33%
0.03%
2.39%
C
control
cu
Percentage of no GFP cells labeled by annexinV-PE
***
coro1a
mpx
lyz

Fig.S14

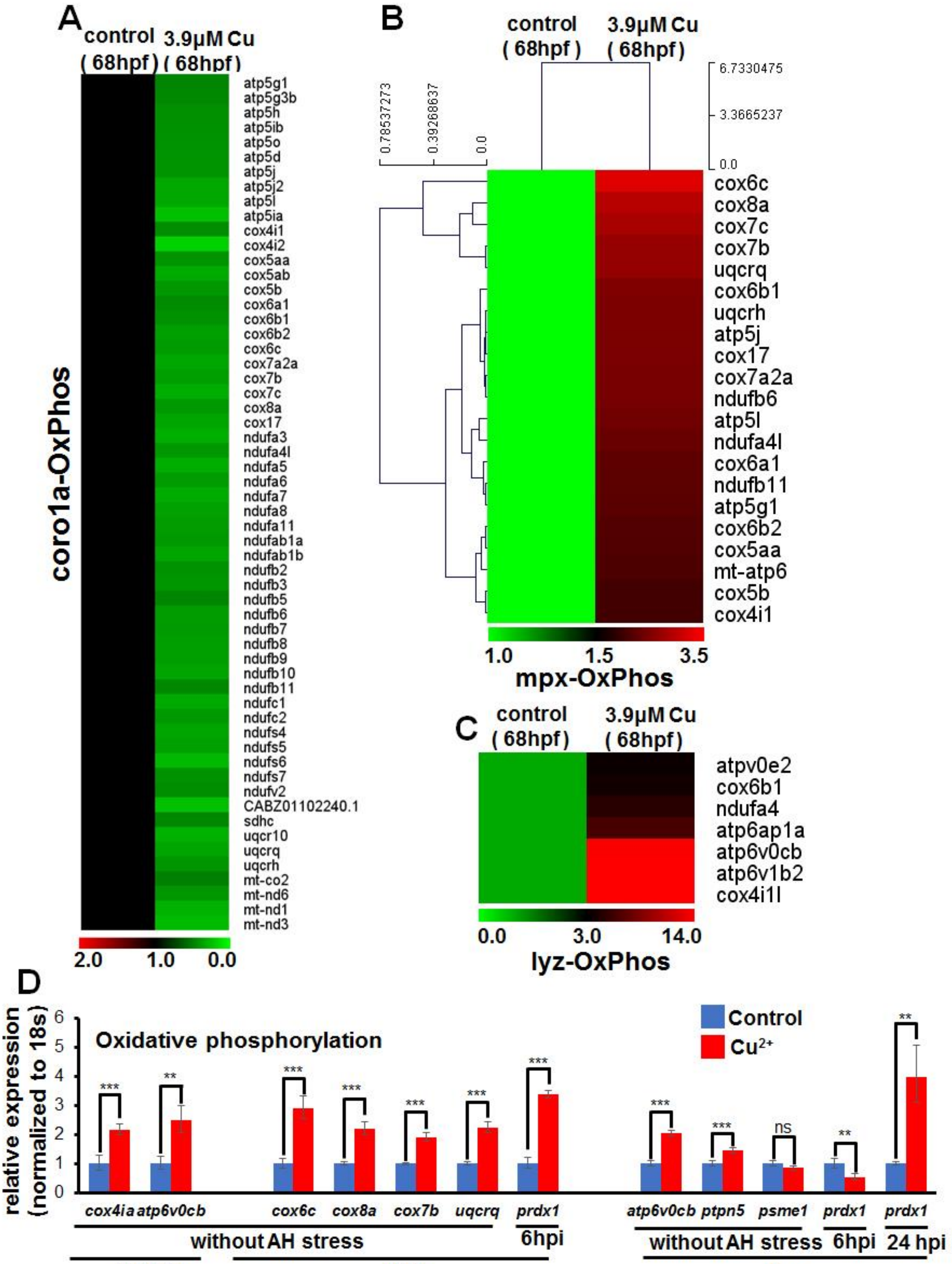

A
control (68hpf)
3.9μM Cu (68hpf)
atp5g1
atp5g3b
atp5h
atp5ib
atp5o
atp5d
atp5j
atp5j2
atp5l
atp5ia
cox4i1
cox4i2
cox5aa
cox5ab
cox5b
cox6a1
cox6b1
cox6b2
cox6c
cox7a2a
cox7b
cox7c
cox8a
cox17
ndufa3
ndufa4l
ndufa5
ndufa6
ndufa7
ndufa8
ndufa11
ndufab1a
ndufab1b
ndufb2
ndufb3
ndufb5
ndufb6
ndufb7
ndufb8
ndufb9
ndufb10
ndufb11
ndufc1
ndufc2
ndufs4
ndufs5
ndufs6
ndufs7
ndufv2
CABZ01102240.1
sdhc
uqcr10
uqcrq
uqcrh
mt-co2
mt-nd6
mt-nd1
mt-nd3
2.0 1.0 0.0
coro1a-OxPhos
B
control (68hpf)
3.9μM Cu (68hpf)
6.7330475
3.3665237
0.0
0.78537273
0.39268637
0.0
cox6c
cox8a
cox7c
cox7b
uqcrq
cox6b1
uqcrh
atp5j
cox17
cox7a2a
ndufb6
atp5l
ndufa4l
cox6a1
ndufb11
atp5g1
cox6b2
cox5aa
mt-atp6
cox5b
cox4i1
1.0 1.5 3.5
mpx-OxPhos
C
control (68hpf)
3.9μM Cu (68hpf)
atpv0e2
cox6b1
ndufa4
atp6ap1a
atp6v0cb
atp6v1b2
cox4i1l
0.0 3.0 14.0
lyz-OxPhos
D
relative expression (normalized to 18s)
Oxidative phosphorylation
Control
Cu2+
cox4ia
atp6v0cb
cox6c
cox8a
cox7b
uqcrq
prdx1
atp6v0cb
ptpn5
psme1
prdx1
prdx1
without AH stress
6hpi
without AH stress
6hpi
24 hpi
coro1a
mpx
lyz

Fig.S15

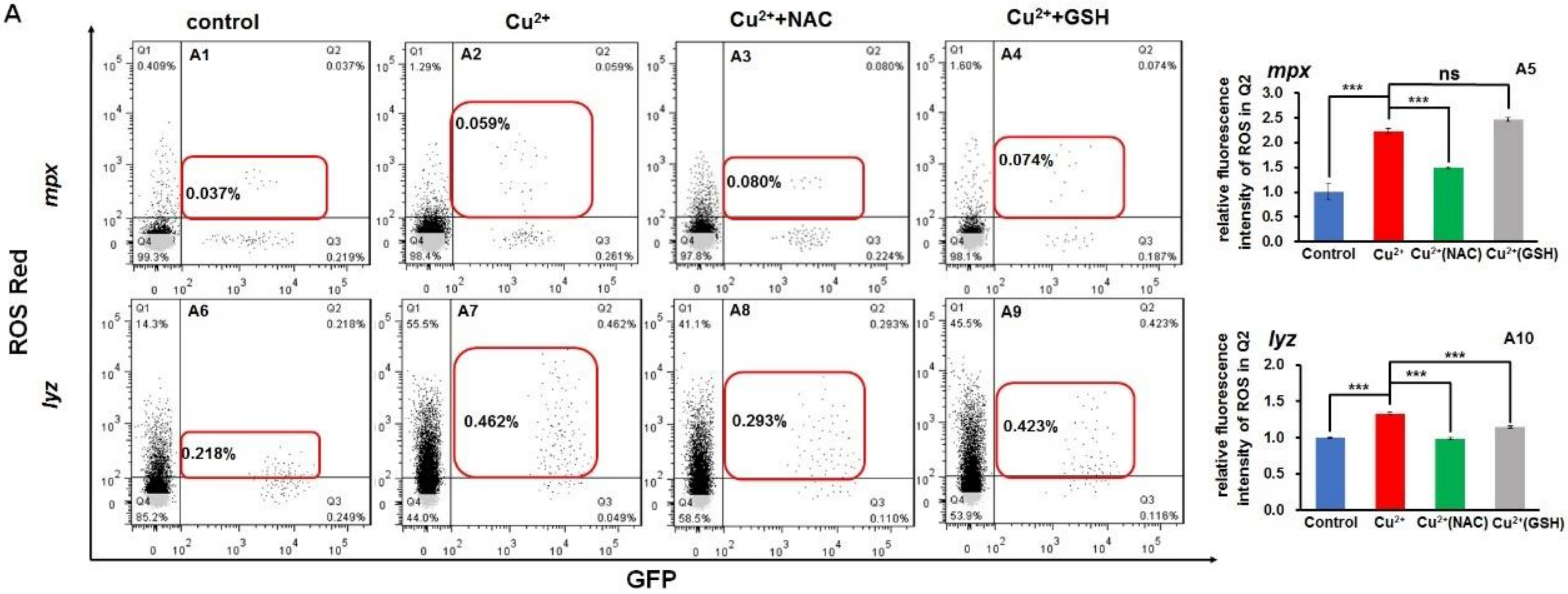
A
control
Cu2+
Cu2++NAC
Cu2++GSH
A1
A2
A3
A4
A5
A6
A7
A8
A9
A10
0.037%
0.059%
0.080%
0.074%
0.218%
0.462%
0.293%
0.423%
mpx
lyz
ROS Red
GFP
relative fluorescence intensity of ROS in Q2
Control
Cu2+
Cu2+(NAC)
Cu2+(GSH)
***
ns

Fig.S16

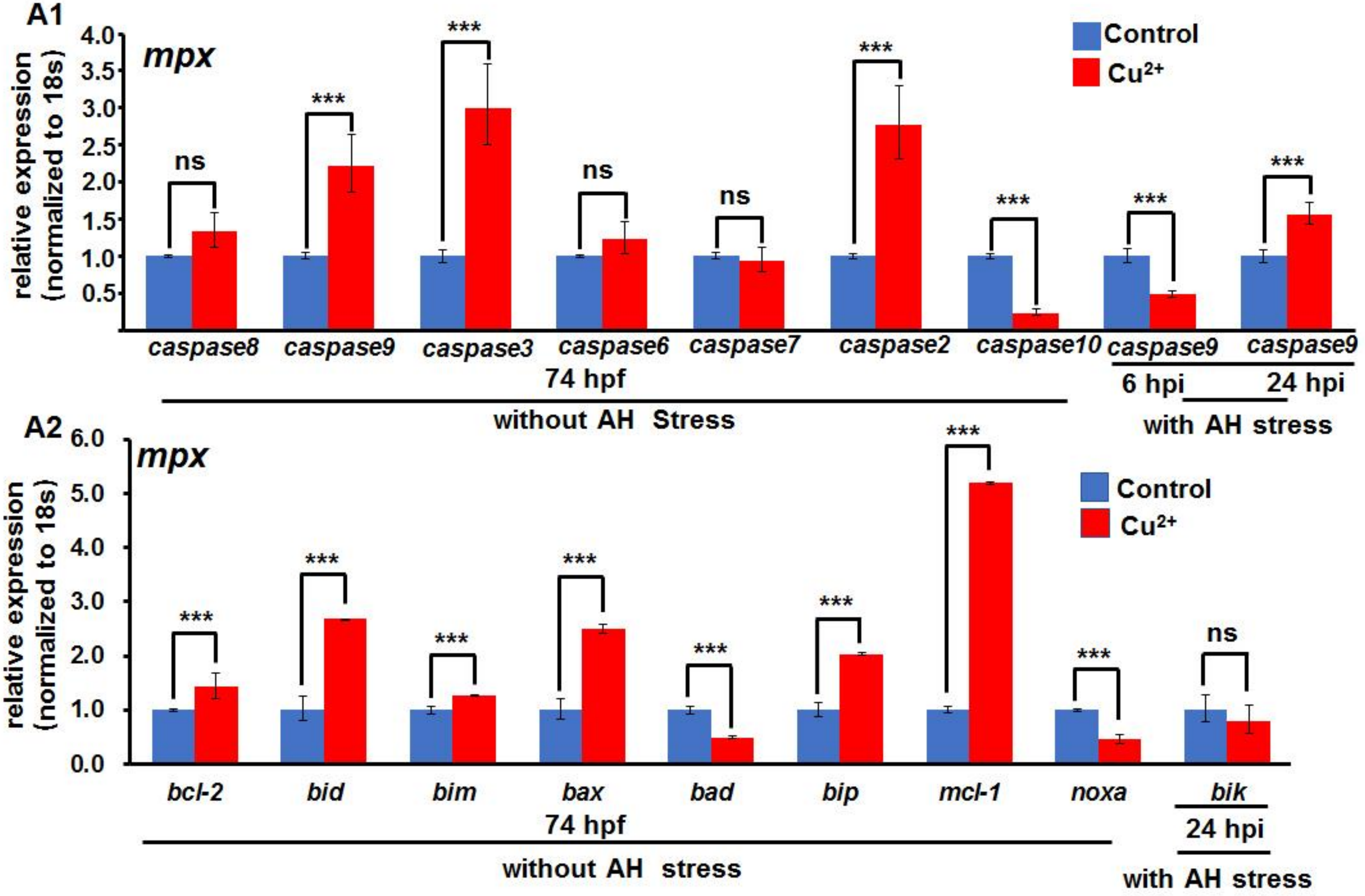
A1
mpx
relative expression (normalized to 18s)
4.0
3.5
3.0
2.5
2.0
1.5
1.0
0.5
Control
Cu2+
ns
***
***
ns
ns
***
***
***
***
caspase8
caspase9
caspase3
caspase6
caspase7
caspase2
caspase10
caspase9
caspase9
74 hpf
6 hpi
24 hpi
without AH Stress
with AH stress
A2
mpx
relative expression (normalized to 18s)
6.0
5.0
4.0
3.0
2.0
1.0
0.0
Control
Cu2+
***
***
***
***
***
***
***
***
ns
bcl-2
bid
bim
bax
bad
bip
mcl-1
noxa
bik
74 hpf
24 hpi
without AH stress
with AH stress

Fig.S17

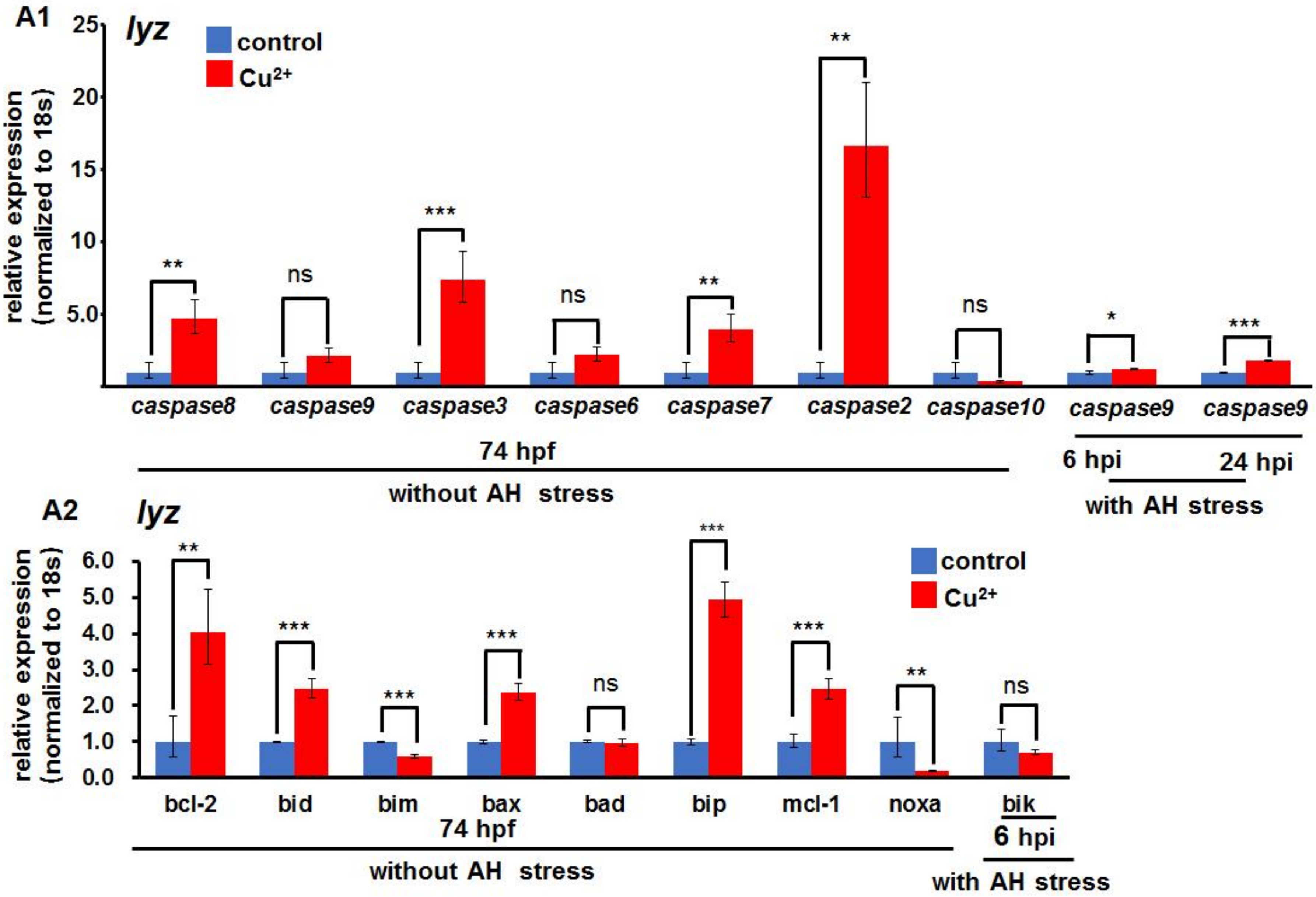
A1
lyz
control
Cu2+
relative expression (normalized to 18s)
25
20
15
10
5.0
**
ns
***
ns
**
**
ns
*
***
caspase8
caspase9
caspase3
caspase6
caspase7
caspase2
caspase10
caspase9
caspase9
74 hpf
without AH stress
6 hpi
24 hpi
with AH stress
A2
lyz
control
Cu2+
relative expression (normalized to 18s)
6.0
5.0
4.0
3.0
2.0
1.0
0.0
**
***
***
***
ns
***
***
**
ns
bcl-2
bid
bim
bax
bad
bip
mcl-1
noxa
bik
74 hpf
without AH stress
6 hpi
with AH stress

Fig.S18
